# Discovery of LaAlO_3_ as an efficient catalyst for two-electron water electrolysis towards hydrogen peroxide

**DOI:** 10.1038/s41467-022-34884-4

**Published:** 2022-11-25

**Authors:** Jihyun Baek, Qiu Jin, Nathan Scott Johnson, Yue Jiang, Rui Ning, Apurva Mehta, Samira Siahrostami, Xiaolin Zheng

**Affiliations:** 1 Department of Mechanical Engineering, Stanford University, Stanford, CA 94305 USA; 2 Department of Chemistry, University of Calgary, Calgary, AB T2N 1N4 Canada; 3 Stanford Synchrotron Radiation Lightsource, SLAC National Accelerator Laboratory, Menlo Park, CA 94025 USA; 4 Department of Materials Science and Engineering, Stanford University, Stanford, CA 94305 USA

**Keywords:** Electrocatalysis, Hydrogen energy, Electrocatalysis

## Abstract

Electrochemical two-electron water oxidation reaction (2e-WOR) has drawn significant attention as a promising process to achieve the continuous on-site production of hydrogen peroxide (H_2_O_2_). However, compared to the cathodic H_2_O_2_ generation, the anodic 2e-WOR is more challenging to establish catalysts due to the severe oxidizing environment. In this study, we combine density functional theory (DFT) calculations with experiments to discover a stable and efficient perovskite catalyst for the anodic 2e-WOR. Our theoretical screening efforts identify LaAlO_3_ perovskite as a stable, active, and selective candidate for catalyzing 2e-WOR. Our experimental results verify that LaAlO_3_ achieves an overpotential of 510 mV at 10 mA cm^−2^ in 4 M K_2_CO_3_/KHCO_3_, lower than those of many reported metal oxide catalysts. In addition, LaAlO_3_ maintains a stable H_2_O_2_ Faradaic efficiency with only a 3% decrease after 3 h at 2.7 V vs. RHE. This computation-experiment synergistic approach introduces another effective direction to discover promising catalysts for the harsh anodic 2e-WOR towards H_2_O_2_.

## Abstract (web summary)

Hydrogen peroxide production from water electrochemical oxidation is a challenging process. Here the authors report discovery of LaAlO3 after screening a series of perovskites as active, stable, and selective catalyst for electrochemical H2O2 production from water oxidation.

## Introduction

Hydrogen peroxide (H_2_O_2_) is an environmentally benign and powerful chemical oxidizer widely used in electronics manufacturing^1,2^, chemical synthesis^3,4^, water purification^5^, and many other industrial sectors. Currently, H_2_O_2_ is produced by the anthraquinone process, which necessitates an energy-demanding multi-electron process and massive infrastructures to transport it to the point of use^6^. Recently, electrochemical processes, such as two-electron oxygen reduction reaction (2e-ORR) and water oxidation reaction (2e-WOR)^7–9^, have emerged as attractive routes to produce H_2_O_2_ onsite in small-scale distributed units^10^. The 2e-ORR has been extensively investigated, and many suitable catalysts with high activity and selectivity have been reported, including noble metals^10–12^, carbon materials^13,14^, and transition metals^15,16^. Unlike 2e-ORR which needs oxygen, the 2e-WOR only requires water as a reactant, thus simplifying the system for the synthesis of H_2_O_2_. The pioneering research on H_2_O_2_ production via 2e-WOR in 2004 showed the feasibility of this process using a carbon-based catalyst in the alkaline electrolyte^17^. Most recent reports on the 2e-WOR catalysts have focused on using metal oxides^18–24^, doped metal oxides^25,26^, heterostructure^27,28^, carbon-based materials^29–31^, and electrolyte engineering^32,33^. However, the 2e-WOR still needs significant improvement in electrocatalysts to be more stable, active, and selective.

The elementary steps for the 2e-WOR are associated with the interaction between the catalyst surface and oxygen intermediates: *O, *OH, and *OOH^34^. For a catalyst to be selective for the 2e-WOR, the binding energy of those intermediates should be in the right range. If the binding is too strong, the adsorbed OH* is further oxidized to *O and *OOH, facilitating 4e-WOR for O_2_ evolution. If the binding is too weak, it is difficult to activate the water molecule and form the *OH. Currently, for 2e-WOR, the state-of-the-art metal oxide catalysts still show more than 1 V overpotential from the equilibrium potential for H_2_O_2_ (1.76 V vs. RHE) to reach 10 mA cm^−2^ of the current density^18,19,21,25^.

Recently, perovskite oxides have garnered significant attention in numerous applications, such as electronics^35^, energy conversion and storage^36^, and catalysts^37,38^. Perovskites have a unit formula ABO_3_, where A cation sites are usually rare-earth or alkaline earth metal, and B cations are typically small transition metal elements staying at the centre of the oxygen octahedron^39^. Perovskites incorporate a broad combination of A and B cations, using more than 90% of metallic elements in the periodic table^39^. As such, perovskites provide rich opportunities to tune their chemical, physical, and catalytic properties.

A superior catalyst for 2e-WOR must meet the following three requirements: (i) high stability to fulfil the demand for long-term operation; (ii) high selectivity to prevent the formation of by-products; (iii) high activity to save energy input. We investigated the stability through a computational screening of a vast number of perovskites in the Materials Project database^40^ as suggested by Persson et al.^41^ This approach has proven to be powerful in rapidly excluding a variety of catalyst materials that do not fall within the desired stability target for long term and practical application. This approach was first introduced by Persson et al. to discover synthesizable and robust photocatalysts for CO_2_ reduction reactions via computational analysis^42^. The recent work by Gunasooriya and Nørskov is another example of applying this approach to discovering stable oxides for the oxygen reduction reaction^43^. Following this approach, we study an extensive library of more than 2000 perovskites to identify 32 stable perovskites at pH = 8 and 10 stable perovskites at pH = 11, conditions relevant for 2e-WOR. Among the 32 stable perovskites, LaAlO_3_ stands out as the most stable perovskite from this study. We further study the activity and selectivity of a series of four other Lanthanum-based (La-based) perovskites including LaCuO_3_, LaNiO_3_ LaGaO_3_, and LaZnO_3_ using density functional theory (DFT) calculation and reported data in the literature^44,45^. We show that LaAlO_3_ is not only the most stable but also the most active and selective catalyst for 2e-WOR among other stable La-based perovskites. It is also the most active, selective, and stable material reported so far^8,18,19,21,25,46^. To verify the computational prediction, we synthesize LaAlO_3_ thin films on FTO glass using the solution-combustion method. The synthesized LaAlO_3_ thin film is confirmed to have a cubic perovskite crystal structure with the dominant phases of (110) and (111) through transmission electron microscopy (TEM), grazing-incidence X-ray diffraction (GIXRD), and X-ray photoelectron spectroscopy (XPS) analysis. Moreover, the LaAlO_3_ thin film has excellent catalytic properties towards 2e-WOR, with an onset potential of 510 mV at 10 mA cm^−2^, a peak faradaic efficiency (FE) of 87% at 3.34 V vs. RHE, H_2_O_2_ production rates of 0.16 mmol cm^−2^ (128 ppm) and 1.23 mmol cm^−2^ (808 ppm) at 2.7 and 3.2 V vs. RHE after 3 h, and good stability over 3 h of testing, supporting the theoretical results.

## Results and discussion

### Computational analysis

**Fig. 1:**
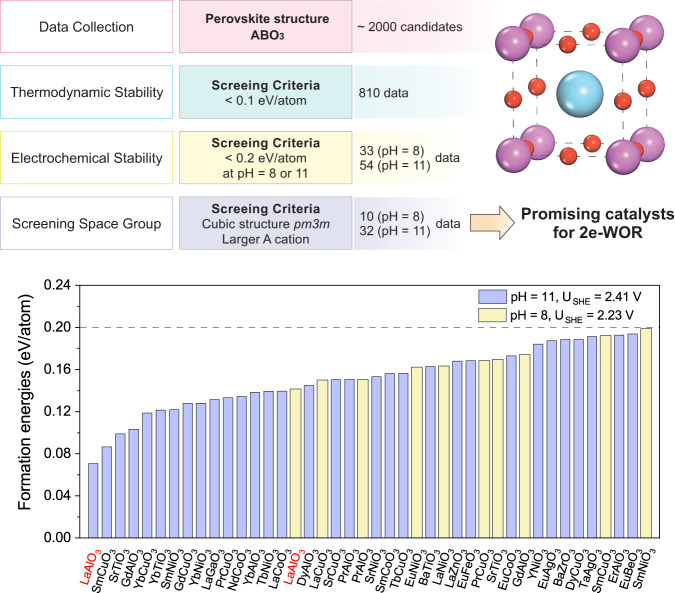
Computational screening to discover the most stable perovskite oxide under different pH conditions. Filtering stable ABO_3_ perovskite structures based on three screening criteria including decomposition energy < 0.1 eV and electrochemical stability under pH = 8 and 11.

As discussed above, stability is a major challenge for catalysts under the harsh anodic oxidation potential conditions required for 2e-WOR toward H_2_O_2_ production. Under highly oxidizing 2e-WOR conditions, lack of catalyst stability is a major cause of catalyst degradation, resulting in a loss of catalytic activity and selectivity^25^. Thus, we first place a particular emphasis on filtering the stable perovskites out of more than 2,000 ABO_3_ structures (Fig. 1). We applied three stability criteria. First, the decomposition energy, defined as a difference between the Gibbs free energies of perovskites and the most stable product they are decomposed to in aqueous media^41^, to be <0.1 eV. The selection of the 0.1 eV threshold was based on the recommendation by Persson et al. to assure the highest stability for perovskites^47^. The second criterion is to be stable under electrochemical conditions (pH = 8 and pH = 11 with applied electrode potentials of 2.23 and 2.41 V versus SHE, respectively) using Pourbaix diagram analyzer using Python Material Genome (Pymatgen)^48^ and Materials Project^40^ cooperatively. The pH=8 and pH=11 were chosen because they are the operating conditions in most experimental studies for 2e-WOR^18,29^. The last criterion is only to include structures with cubic ${Pm}\bar{3}m$ space group and a larger A cation as they are the most stable and typical crystal structures for ABO_3_ perovskites. The use of these three criteria yields 10 (Fig. 1 and Supplementary Table 1) and 32 (Fig. 1 and Supplementary Table 2) stable perovskites at pH = 8 and 11, respectively. Among them, LaAlO_3_ was identified as the most stable with the least formation energy at both pH values (pH = 8 and 11). Additionally, the superior stability of LaAlO_3_ has been revealed by the extremely high O-vacancy formation energy of 2.91 eV, as shown in Supplementary Fig. 1. The less stable perovskites with higher formation energies are more likely to suffer from catalyst aging, thereby resulting in catalyst deactivation and the demand of more frequent catalyst replacement in the device to maintain a target level of H_2_O_2_ production. We note that the stability analysis based on the Pourbaix diagram analyzer, introduced by Persson et al.^41^ is established based on sole thermodynamic analysis with no consideration of the possible kinetic stability as a result of the slow dissolution process or formation of resistant films on the catalyst surface under reaction conditions. Previous studies show that materials such as BiVO_4_^25^ for the 2e-water oxidation reaction that does not come out as a stable material from the Pourbaix diagram analysis can survive the anodic reaction conditions for several hours. On the other hand, approaches can be taken to increase the stability of those materials. For example, doping BiVO_4_ with Gadolinium has been used as a strategy to improve the long-term stability of BiVO_4_ by preventing the leaching of vanadate ions^25^. In this work, we used the Pourbaix Diagram analysis to mark the materials that inherently have high stability, not taking into account the ones that may not be thermodynamically stable but may show kinetic stability as a result of a slow dissolution process under reaction conditions or those that may make a resistant film under reaction conditions. Moreover, since LaAlO_3_ is the most stable perovskite under 2e-WOR conditions, it is highly promising to eliminate the effect of catalyst degradation, maintain catalytic performance and extend its stability to meet the long-term operation targets. Apart from the exceptional stability, LaAlO_3_ is earth-abundant and non-toxic, providing a potential to investigate the 2e-WOR activity for LaAlO_3_, which will be described in the later section.

**Fig. 2:**
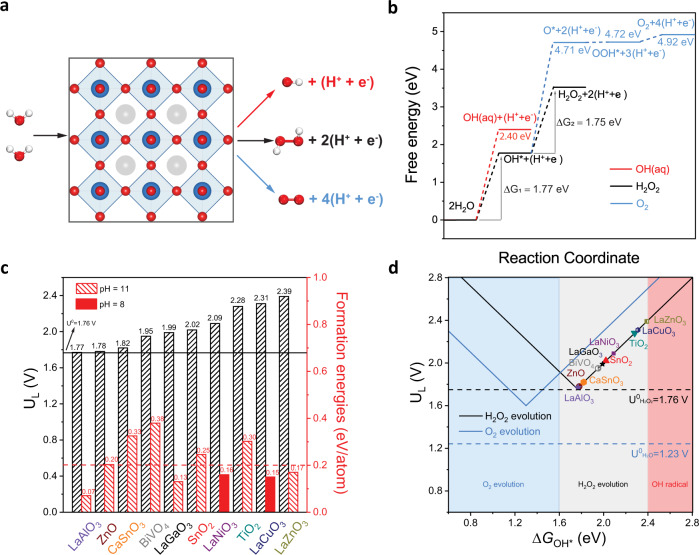
Mechanism of the 2e-WOR H_2_O_2_ production in LaAlO_3_ and comparison of limiting potential and formation energy with different catalysts. **a** WOR processes via different pathways. La, Al, O, and H are represented by gray, blue, red, and white spheres, respectively. **b** Free energy diagram of all the three WORs over LaAlO_3_. **c** Calculated limiting potentials (U_L_, left axis) for the metal oxides, and the corresponding formation energies (right axis) under 2e-WOR (electrolyte pH = 11 or 8). **d** Volcano plots of limiting potential as a function of Δ*G*_OH*_.

Next, we employed DFT calculation to evaluate the theoretical activity and selectivity of LaAlO_3_ for 2e-WOR. Water oxidation may follow four-electron, two-electron, and one-electron pathways (Fig. 2a); hence selectivity toward H_2_O_2_ product is a great challenge. Previous studies have identified that the binding energies of different reaction intermediates (O*, OH*, and OOH*) are suitable descriptors for the activity and selectivity of water oxidation reaction pathways^7,8^. An active catalyst for 2e-WOR has an optimum OH* binding (Δ*G*_OH*_ ≥ 1.76 eV with reference to H_2_O). In order to be selective, the catalyst should weakly bind O* (Δ*G*_O*_ ≥ 3.52 eV) to suppress the 4e-WOR route and prevent the further oxidation of H_2_O_2_ to O_2_. Moreover, the catalyst should bind OH* below 2.4 eV (Δ*G*_OH*_ < 2.4 eV) to avoid the formation and release of OH radicals. For LaAlO_3_ (100) surface, the adsorption energies of these critical intermediates are Δ*G*_OH*_ = 1.77 eV and Δ*G*_O*_ = 4.71 eV (Supplementary Fig. 2). Thus, LaAlO_3_ is highly selective and active for the 2e-WOR and suppresses both the 1e- and the 4e-WOR pathways, as shown in Fig. 2b. Note that the (100) facet of LaAlO_3_ was used in this analysis, as it is predicted as the most stable among (100), (110), and (111) facets based on surface energy calculations (Supplementary Fig. 3).

In addition to LaAlO_3_, we investigated the 2e-WOR of four other La-based perovskites that were identified as stable materials in Fig. 1. We performed explicit calculations for LaCuO_3_ and LaNiO_3_ (Supplementary Fig. 4) and data for LaGaO_3_ and LaZnO_3_ were extracted from the literatures^44,45^. The calculated limiting potential (U_L_) and relative stability of LaAlO_3_ are compared in Fig. 2c to four other La-based perovskites as well as several oxides that have been evaluated for 2e-WOR^18,19^. The solid upper line marks the thermodynamic potential of 1.76 V for 2e-WOR. The lower dashed line indicates the electrochemical stability with a formation energy of 0.2 eV/atom. Figure 2c shows that LaAlO_3_ is the most active La-based perovskite (with the lowest calculated limiting the potential of 1.77 eV) and the most stable (pH = 11, results for pH = 8 in Supplementary Fig. 5) catalyst among previously reported oxides under 2e-WOR conditions. Figure 2d shows the U_L_ vs. Δ*G*_OH*_ for those oxides and perovskites. The binding of OH* on the LaAlO_3_ surface is 1.77 eV which is the closest to the optimal value of 1.76 eV. In addition, we performed the Bader charge analysis to understand the active site in LaAlO_3_ (Supplementary Fig. 6). Our results revealed a strong electron-accepting feature on the Al sites, characterized by higher charges (+2.41 to +2.43 e) as compared to the ones on La (+2.05 to +2.07 e) coming from its oxophilic nature. These more positive charges can facilitate the adsorption of negatively charged OH species in the alkaline electrolyte and thereby increase the activity of LaAlO_3_ toward 2e-WOR. To understand the contribution of O-vacancies and defects in the activity of LaAlO_3_, we calculated the OH* adsorption on LaAlO_3_ with O-vacancy (Supplementary Fig. 1c). The O-vacancies are strongly binding to the OH* intermediate (Δ*G*_OH*_ = −2.51 eV), indicating the O-vacancies do not contribute to the 2e-WOR. Since the formation of additional O-vacancies in LaAlO_3_ is strongly endothermic (by ~3 eV) as stated above, it is unlikely for LaAlO_3_ to form additional defects under 2e-WOR conditions. Thus, according to the DFT results, LaAlO_3_ is the stable, active, and selective catalyst for 2e-WOR.

We note that the carbonate anion has also been considered in the H_2_O_2_ reaction calculation since it is essential in the commonly used electrolyte (2 M KHCO_3_)^18,32,49^. To evaluate the effect of carbonate anion on H_2_O_2_ production, we calculated the 2e-WOR pathway with carbonate anion as a mediator as reported by Fan et al. (Supplementary Fig. 7)^50^. This analysis shows that the largest free energy change via carbonate mediated pathway is 2.03 eV to generate H_2_O_2_, indicating that the theoretical limiting potential is 2.03 V, slightly higher than that of 2e-WOR pathway without carbonate (1.77 V). Additionally, we calculated the H_2_CO_3_ oxidation mechanism proposed by Kusama et al. (Supplementary Fig. 8) on the LaAlO_3_ surface^51^. Our results agree well with the report by them and show that the first proton removal from the adsorbed H_2_CO_3_ is the rate-limiting step. The energy difference we calculated for this step is 1.89 eV, indicating a limiting potential of 1.89 V. This limiting potential of 1.89 V is also slightly (~0.1 V) higher than the value calculated for 2e-WOR on bare LaAlO_3_ (1.77 V). However, all these three values (2.03, 1.77 and 1.89 V) are reasonably close, and under the operating reaction potentials all pathways will be accessible. Thus, we conclude that the carbonate and H_2_CO_3_ oxidation provide additional pathways to promote the H_2_O_2_ production.

### Synthesis of LaAlO_3_ perovskite oxide catalyst

**Fig. 3:**
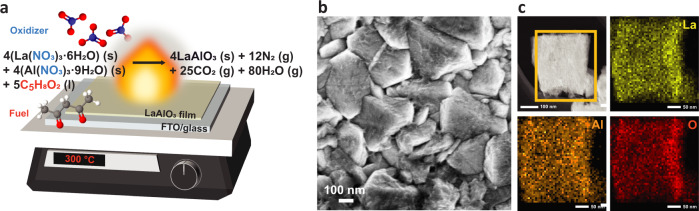
Solution combustion synthesis process and characterizations of the final product. **a** Schematic of low-temperature solution combustion processing mechanism for LaAlO_3_ synthesis. **b** SEM top-view and **c** TEM dark-field and EDS mapping images of the synthesized LaAlO_3_.

The synthesis of LaAlO_3_ perovskite oxide requires a high temperature of 800–1700 ˚C to obtain a pure cubic crystal structure with a space group of ${Pm}\bar{3}m$, and LaAlO_3_ has been synthesized by methods such as the solid-state reaction of Al_2_O_3_ and La_2_O_3_^52,53^, co-precipitation^54^, aerosol technique^55^, and sol-gel method^56,57^. To remove the need for such a high-temperature source, we employed a self-sustaining solution-based combustion synthesis method^58,59^ to prepare LaAlO_3_ that requires heating only at 300 ˚C. As illustrated in Fig. 3a, acetylacetone (C_5_H_8_O_2_) and metal nitrates serve as fuel and oxidizers, and their reaction onset temperature is about 240 ˚C as determined by the thermogravimetric analysis (TGA) and differential scanning calorimeter (DSC) measurement (Supplementary Fig. 9). The mixture of metal nitrates and acetylacetone was spin-coated on an FTO glass, and we set the hot plate at 300 °C to have sufficient temperature to initiate their exothermic reaction, and the generated heat enabled the conversion of the metal precursors to LaAlO_3_ and other gaseous products. The synthesis conditions, in terms of the precursor ratio, processing temperature, and the number of coating layers on the activity, are summarized in Supplementary Fig. 10. Fig. 3b shows the top-view scanning electron microscopy (SEM) image of as-synthesized LaAlO_3_ on the FTO glass. The bare FTO has grains of several hundred nanometers scale with smooth surfaces. After the LaAlO_3_ film is deposited on top, the FTO glass looks similar, but the grain surface looks rougher and more wrinkled, which looks like 2D sheets with a thickness of roughly 20–30 nm (Supplementary Fig. 11). The transmission electron microscopy (TEM) dark-field image and energy-dispersive X-ray spectroscopy (EDS) elemental analysis in Fig. 3c and SEM-EDS mapping images on several spots throughout the film in Supplementary Fig. 11 show that all elements (La, Al, and O) are uniformly distributed throughout the whole area of the film.

### Material characterizations of LaAlO_3_ perovskite

**Fig. 4:**
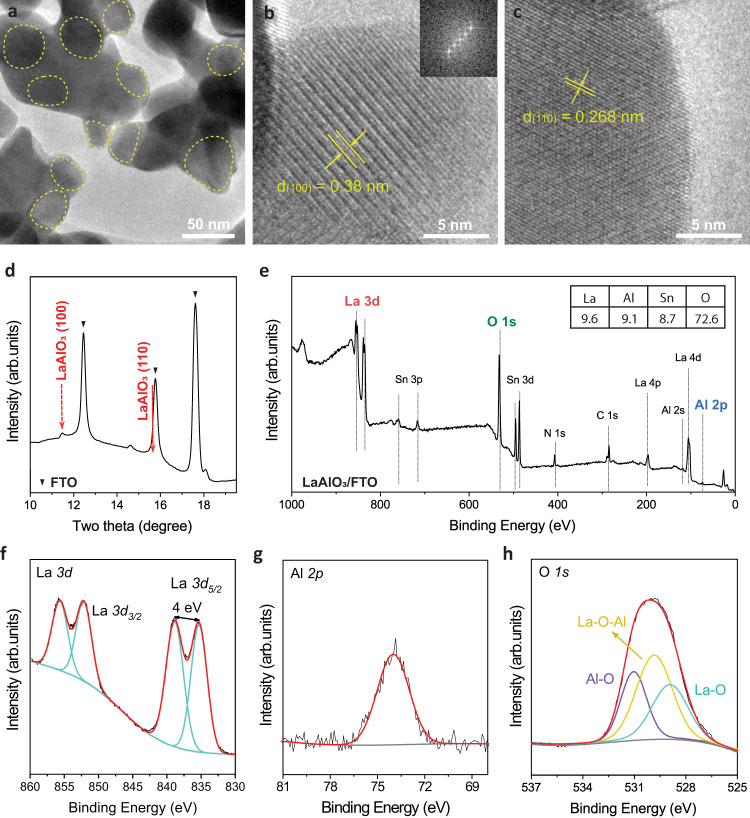
Key characterizations of the synthesized LaAlO_3_. **a**–**c** High-resolution TEM images of LaAlO_3_ synthesized by sol-gel combustion method. From the TEM images, the morphology shows an irregular shape with the size of several tens of nm. And most of the parts look amorphous, while a crystalline LaAlO_3_ phase was created at some surface parts. This resulted from the temperature spontaneously attained during the combustion process instantaneously that extends to the range of 2000 °C for short periods. **d** GIXRD result of LaAlO_3_ on FTO/glass substrate, **e** Surface analysis using XPS survey spectra with the inlet table with the elemental concentration (%), and the detailed elemental regions: **f** La 3d, **g** Al 2p, and **h** O 1 s.

High-resolution TEM (HRTEM) images of LaAlO_3_ in Fig. 4a reveal that the particles are composed of both amorphous and crystalline parts (yellow circles). As shown in Fig. 4b, c, and Supplementary Fig. 12, HRTEM images show that the cubic structure with a space group of ${Pm}\bar{3}m$ of the LaAlO_3_ phase belongs to the (100) and (110) planes, which has a lattice spacing of 3.8 and 2.7 Å, respectively. Moreover, these planes in ${Pm}\bar{3}m$ space group have formation energies of 0.11 and 0.28 eV/atom, which is favorable for 2e-WOR from the DFT calculations in Supplementary Fig. 3. In addition to the TEM analysis, the Grazing-incidence X-ray diffraction (GIXRD) measurement in Fig. 4d shows that the formed crystalline particles on FTO have the LaAlO_3_ perovskite structure (ICDD 01–083–4238), for which the peak at 11.46 ° corresponds to the (100) plane. The 15.7° peak is overlapped by the (110) peak of LaAlO_3_ and the FTO substrate peak; however, the HRTEM confirms the (110) facet for LaAlO_3_. Since the GIXRD was collected with the X-rays with its incident energy of 17.0 keV and wavelength of 0.729 Å^60^, the two theta values are different from those with the general Cu Kα (1.54 Å). All the above results confirm that the crystal structure of LaAlO_3_ is cubic perovskite.

In addition, X-ray photoelectron spectroscopy (XPS) was performed to investigate the surface elemental components of the as-synthesized LaAlO_3_. The XPS survey profile in Fig. 4e shows that the elemental ratio between La and Al was approximately 1. The high-resolution XPS spectra in Fig. 4f show that the La *3d* core level splits into *3d*_*3/2*_ and *3d*_*5/2*_ components due to the spin-orbit coupling. The observed La *3d*_*5/2*_ doublet has a splitting of ~4.0 eV, which agrees with the formation of lanthanum oxide^61,62^. In comparison, the sol-gel synthesized LaAlO_3_ has a *La 3d*_*5/2*_ doublet of about 3.7 eV, which indicates lanthanum hydroxide species (Supplementary Fig. 13). Furthermore, the O 1 s core level for combustion-synthesized LaAlO_3_ in Fig. 4h consists of three peaks: La–O, La–O–Al, and Al–O at 529 eV, 530.1 eV, and 531.2 eV, respectively^63–65^. In another way, the deconvoluted O *1s* peak also contains a slight amount of oxygen vacancies in the lattice and the ratio between the oxide and oxygen vacancy is approximately 10:1 based on their integrated areas, which can contribute to the conductivity of LaAlO_3_ (Supplementary Fig. 14). The O *1* *s* peaks for sol-gel synthesized LaAlO_3_ imply that the surface contains the hydroxide species rather than the oxides (Supplementary Fig. 13). Those analyses indicate that the solution-based combustion reaction effectively converts the metal precursors into crystalline perovskite oxide^58,66^.

### Evaluation of electrocatalytic H_2_O_2_ evolution properties on LaAlO_3_ perovskite oxide

**Fig. 5:**
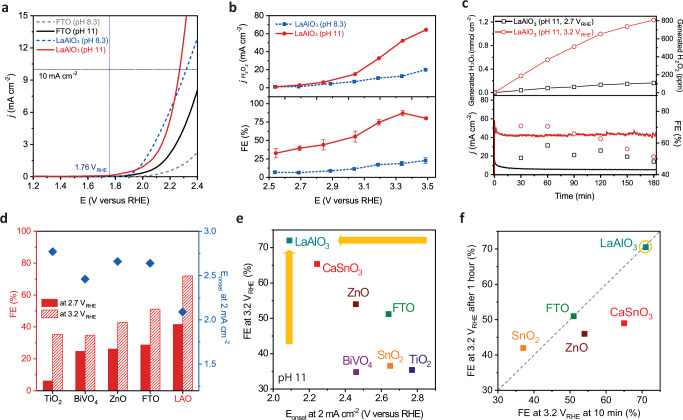
Electrochemical performance. **a** Linear sweep voltammetry curves of LaAlO_3_ anode compared to FTO/glass substrate in the electrolyte of 2 M KHCO_3_ (pH 8.3) and 4 M K_2_CO_3_/KHCO_3_ (pH 11). All curves are 95 % iR-compensated. **b** The calculated H_2_O_2_ current density and selectivity of LaAlO_3_ anode as a function of potential in different electrolytes, and **c** the generated H_2_O_2_ amount, selectivity, and stability of the LaAlO_3_ anode at different potentials, 2.7 and 3.2 V vs. RHE for 3 h. **d** Comparison of Faradaic efficiencies at different potentials and onset potentials to reach 2 mA cm^−2^ with the previously reported materials in pH 11. **e** Selectivity vs. activity plot for different metal oxides in the electrolyte of 4 M K_2_CO_3_/KHCO_3_ (pH 11) at 3.2 V vs. RHE. **f** Comprehensive plot for selectivity and stability in the electrolyte of 4 M K_2_CO_3_/KHCO_3_ (pH 11) at 3.2 V vs. RHE. The *x*-axis is the FE at time *t* = 10 min. The *y*-axis is the FE at time *t* = 1 h. The diagonal dashed line is the expected response for a stable catalyst.

Figure 5a plots the linear sweep voltammetry (LSV) curves of the combustion-synthesized LaAlO_3_ on FTO/glass and the bare FTO/glass in two different electrolytes: 2 M KHCO_3_ (pH = 8.3) and 4 M K_2_CO_3_/KHCO_3_ (molar ration of 1:7, pH = 11). The 2 M KHCO_3_ is the commonly used electrolyte for 2e-WOR^18,32,49^, while the 4 M K_2_CO_3_/KHCO_3_ electrolyte was recently identified as a better electrolyte with higher selectivity towards 2e-WOR^33,67^. Indeed, both LaAlO_3_/FTO and bare FTO exhibit earlier onset potentials in the newly optimized 4 M K_2_CO_3_/KHCO_3_ electrolyte. The overpotentials at 10 mA cm^−2^ of LaAlO_3_/FTO film were measured to be 560 mV in 2 M KHCO_3_ and 510 mV in 4 M K_2_CO_3_/KHCO_3_ (equilibrium potential: 1.76 V vs. RHE). In both electrolytes, the overpotentials of LaAlO_3_/FTO are smaller than those of bare FTO. The electrochemical impedance spectroscopy (EIS) analysis was conducted to compare the conductivity of LaAlO_3_ and FTO substrate at 2.7 V vs. RHE (Supplementary Fig. 15). The data were fitted with a REAP2CPE equivalent circuit model in which there were three types of resistances: resistance in the electrolyte solution (R1), charge transfer resistance inside bulk catalyst (R2), and charge transfer resistance at the interface between the catalyst and electrolyte (R3). Here, we could emphasize R2 and R3 values, which contribute to the conductivity inside the bulk of the catalyst material and at the interface between the material surface and the electrolyte. Here, LaAlO_3_ film shows roughly 3.5 times lower bulk and interface resistances than FTO itself at 2.7 V vs. RHE, which is the desired potential for H_2_O_2_ production. This presumably results from the oxygen vacancy in the lattice (Supplementary Fig. 14). This can also be evidence that our LaAlO_3_ is not an insulating material compared to FTO. In addition, we also measured the activity for some stable perovskite oxides, based on the formation energies (Fig. 1), in 4 M K_2_CO_3_/KHCO_3_ (pH 11; Supplementary Fig. 16). All perovskite oxides here were synthesized by the same method as that for LaAlO_3_. We found that SmCuO_3_ and GdCuO_3_ also showed relatively good activity (610 and 660 mV at 10 mA cm^−2^, respectively) but were still inferior to the activity of LaAlO_3_. Figure 5b shows that the H_2_O_2_ current densities (j_H2O2_) and the Faradaic efficiencies (FE) of LaAlO_3_/FTO are higher in 4 M K_2_CO_3_/KHCO_3_ (pH 11) than those in 2 M KHCO_3_ (pH 8.3), especially at higher bias, and the peak FE reaches 87 % at 3.34 V vs. RHE in the optimized electrolyte vs. 23 % at 3.5 V vs. RHE in 2 M KHCO_3_ (see Methods section for details). We further tested the stability of LaAlO_3_/FTO in 4 M K_2_CO_3_/KHCO_3_ under two current densities of 3 and 33 mA cm^−2^ at 2.7 and 3.2 V vs. RHE, respectively. Figure 5c shows that the LaAlO_3_/FTO has a smaller FE decrease at 2.8 V vs. RHE (from 51% to 48%) than at 3.2 V vs. RHE (70% to 51%) after 3 h of the test. After 3 h of the durability test, 0.16 mmol cm^−2^ (128 ppm) and 1.23 mmol cm^−2^ (808 ppm) of H_2_O_2_ were generated at 2.7 and 3.2 V vs. RHE, respectively, in comparison to only 70 μmol cm^−2^ at 2.7 V vs. RHE for bare FTO (Supplementary Fig. 17). After the stability test, we measured the post-situ XPS and SEM images to assess any elemental composition and morphology changes (Supplementary Figs. 18 and 19). No compositional degradations in La *3d*, Al *2p*, and O *1* *s* regions were observed from the post-situ XPS analysis after 3 h of the durability test nor any morphological degradation from the SEM images.

Figure 5d compares the FEs (right) at 2.7 and 3.2 V vs. RHE of LaAlO_3_/FTO and the onset potential to attain 2 mA cm^−2^ (left) with previously reported metal oxide catalysts on FTO in the same electrolyte (4 M K_2_CO_3_/KHCO_3_). The LaAlO_3_ perovskite oxide exhibits much higher FEs and lower onset potential than TiO_2_, BiVO_4_, ZnO, and FTO. Additionally, LaAlO_3_ has the lowest overpotential to reach 2 mA cm^−2^ among all the previously reported metal oxides in 2 M KHCO_3_ in Supplementary Fig. 20. Furthermore, we conducted the activity, stability, and selectivity test for some of the state-of-the-art metal oxides (CaSnO_3_, ZnO, and SnO_2_) to compare with those for LaAlO_3_ in 4 M K_2_CO_3_/KHCO_3_ (pH 11), showing that LaAlO_3_ exhibited the lower overpotential, higher FEs and generated H_2_O_2_ amount (Supplementary Fig. 21). Other oxides (BiVO_4_, TiO_2_, ZnO, and FTO) were investigated for electrochemical H_2_O_2_ production under the same condition in our previous study^32,67^. In Supplementary Fig. 22 (Reprinted (adapted) with permission from ref. 68. Copyright 2021 American Chemical Society.)^68^, the production rate of H_2_O_2_ for LaAlO_3_ is much higher than other reported metal oxides and similar to the boron-doped diamond (BDD). Finally, we assessed different state-of-the-art metal oxides by plotting FEs at 3.2 V_RHE_ vs. onset potential to reach 2 mA cm^−2^ in 4 M K_2_CO_3_/KHCO_3_ (pH 11) in Fig. 5e. LaAlO_3_ is positioned at the very upper left side of the plot, which means the most active and selective catalyst among different metal oxides. Figure 5f also demonstrates the comprehensive plot for the selectivity and stability in 4 M K_2_CO_3_/KHCO_3_ (pH 11) at 3.2 V vs. RHE. The *x*-axis is the FE at *t* = 10 min, and the *y*-axis is the FE at *t* = 1 h. The diagonal dashed line is the expected response for a stable catalyst. LaAlO_3_ is located on both the expected response line and the highest FE region, which indicates the most selective and stable catalyst among the known state-of-the-art metal oxides. These results indicate that the LaAlO_3_ catalyst has superior activity and selectivity for 2e-WOR to produce H_2_O_2_, especially in the recently optimized 4 M K_2_CO_3_/KHCO_3_ electrolyte.

Here, we utilized combined computational screening and experimental results to identify LaAlO_3_ as the most stable, active, and selective catalyst reported thus far for 2e-WOR to H_2_O_2_ production. First, we extensively investigated a library of more than 2000 perovskites to identify 32 stable perovskites at pH=8 and 10 stable perovskites at pH = 11 for 2e-WOR. Among them, LaAlO_3_ was found as the most stable with the smallest formation energy under both pH conditions. Then, in order to provide a potential to examine the 2e-WOR activity, we synthesized LaAlO_3_ via self-sustaining solution-combustion method. The synthesized LaAlO_3_ exhibits a cubic crystal structure with dominant planes of (100) and (110). The catalytic properties of LaAlO_3_ towards 2e-WOR were evaluated at two different electrolytes: 2 M KHCO_3_ (pH 8.3) and 4 M K_2_CO_3_/KHCO_3_ (1:7, pH 11), and LaAlO_3_ shows better activity towards 2e-WOR than TiO_2_, BiVO_4_, ZnO, and FTO in both electrolytes. LaAlO_3_ achieves a superior 2e-WOR activity (510 mV of the overpotential to reach 10 mA cm^−2^), excellent selectivity (the peak FE of 87 % at 3.34 V vs. RHE), and stability (only 3 % of the FE degradation after 3 h).

## Methods

### DFT calculations

Density functional theory (DFT) calculations were performed using the Vienna ab initio Simulation Package (VASP) code^69^. The projector augmented wave (PAW) method was used to describe the interactions between electrons and ion cores^70^. The electron exchange and correlation energy were described using the revised Perdew-Burke-Ernzerhof (RPBE) functional^71^. A plane-wave cutoff energy of 500 eV was utilized to expand the wavefunctions. The total energy convergence was set to be lower than 10^−6 ^eV, and the convergence criterion for geometry optimizations was set to a maximum force of 0.02 eV/Å. *U* values of 7.0 and 4.5 eV were set for LaNiO_3_ and LaCuO_3_, respectively, to describe the strong correlation effect of Ni *3d* and Cu *3d* by the DFT + U method. Periodic boundary conditions were used in all directions, and the facets of (100), (110), and (111) for LaAlO_3_ were investigated. A Monkhorst-Pack grid with dimensions of 4 × 4 × 1 was used for sampling the first Brillouin zones^72^, and 15 Å of vacuum in the z-direction was employed to separate the slabs.

The two-electron reaction pathway for WOR can be written as:

$${{{{{{\rm{H}}}}}}}_{2}{{{{{\rm{O}}}}}}+\ast \to*{{{{{\rm{OH}}}}}}+({{{{{{\rm{H}}}}}}}^{+}+{{{{{{\rm{e}}}}}}}^{-})$$

$${{{{{{\rm{H}}}}}}}_{2}{{{{{\rm{O}}}}}}+\ast \to*{{{{{\rm{OH}}}}}}+({{{{{{\rm{H}}}}}}}^{+}+{{{{{{\rm{e}}}}}}}^{-})$$

$$\ast {{{{{\rm{OH}}}}}}+\ast {{{{{\rm{OH}}}}}}\to {{{{{{\rm{H}}}}}}}_{2}{{{{{{\rm{O}}}}}}}_{2}$$

The reaction energy of each elementary step for the WOR has been calculated based on the method developed by Nørskov et al. ^73^ as follows:

1 $$\varDelta G=\varDelta E+\varDelta ZPE-T\varDelta S+\varDelta {G}_{U}+\varDelta {G}_{pH}+\varDelta {G}_{field}$$

2 $$\varDelta {G}_{U}=eU$$

3 $$\varDelta {G}_{pH}=0.0592\times pH$$

where *∆E* is the total energy change obtained from DFT calculations, *∆ZPE* is the change of zero-point energy, *T* is the temperature (298.15 K), and *∆S* represents the entropy change. *U* is the electrode potential (vs. reversible hydrogen electrode), and e^-^ is the charge transferred. *ΔG*_*field*_ is the free energy correction due to the electrochemical double layer and is neglected as suggested in previous studies^73,74^. Zero-point energy and entropies of the adsorbed species were calculated from the vibrational frequencies (Supplementary Table 3). We used the computational hydrogen electrode model, which exploits *G(H*^+^ *+ e*^-^*)* = $\frac{1}{2}$*G(H*_2_*)* under *pH* = 0 and *U* = 0 V. The free energy of H_2_O was calculated from gas-phase H_2_O at 0.035 bar, 298.15 K.

To study the stabilities of (100), (110), and (111) facets for LaAlO_3,_ we calculated their surface energies using the following equation:

4 $${\varUpsilon }=({E}_{tot}-{N}_{La}\ast {E}_{bulk})/{{{{{\rm{N}}}}}}$$

where *E*_*tot*_ and *E*_*bulk*_. refer to the total energies of the structures with a specific facet and unit bulk, N_La_ and ${{\mbox{N}}}$ refers to the numbers of Lanthanum and total atoms, respectively.

### Computational screening

A large library of more than 2000 perovskites has been investigated using high-throughput screening. In the first step, we set the stability criteria to E above hull ≤ 0.1 eV to filter the stable perovskites. Next, we used the Pourbaix diagram to predict the compositions of oxides and identify the electrochemical stability of perovskites under different reaction conditions (various pH and potentials). Pourbaix diagram was constructed by using Python Material Genome (Pymatgen)^48^ and Materials Project^40^.

### Sample preparation

All chemical precursors were purchased from Sigma Aldrich. We deployed the acetylacetone-based sol-gel combustion synthesis. First, 0.2 M of La(NO_3_)_3_·6H_2_O and Al(NO_3_)_3_·9H_2_O were dissolved in 5 ml of 2-methoxyethanol in a 1:1 ratio, followed by 0.2 ml of acetylacetone. After completely dissolving the metal salts, 114 µl of NH_4_OH was added to the solution. Then, the solution was aged for 12 h. Next, the 80 µl of the precursor solution was spin-coated on the cleaned fluorine-doped tin oxide (FTO, 7–8 ohm/sq, MSE supplies) substrate at 3000 rpm for 20 s, then annealed at 300 °C for 30 min under air. Here, a combustion-based synthetic approach is applied to oxide thin films, using acetylacetone as a ‘fuel’ and metal nitrates as oxidizers^58^.

### Materials characterizations

Sample morphology was investigated using scanning electron microscopy (SEM, FEI Magellan 400), and high-resolution, dark-field, and energy-dispersive X-ray spectroscopy (EDS) mapping images were collected by transmission electron microscopy (TEM, FEI Tecnai G2 F20 X-TWIN, 200 kV) at Stanford Nano Shared Facilities (SNSF). The TEM instrument was run using the TIA interface, and Gatan Digital Micrograph was employed to calculate the material d-spacing value with a Fast Fourier Transform (FFT) image. Here, d-spacing was determined by the gap between two symmetrical diffraction points in the FFT image.

Grazing-incidence X-ray diffraction (GIXRD) was measured at Beamline 2-1 of the Stanford Synchrotron Radiation Lightsource at SLAC National Accelerator Laboratory with an incident energy of 17.0 keV (0.729 Å) and a Huber 2-circle goniometer. The beam was sliced down to a vertical height of ~ 30 μm and a nominal width of 1 mm. The scattered X-rays were determined using a Pilatus 100 K area detector from Dectris with 487 × 195 pixels (172 μm × 172 μm pixel size). During measurements, the incident angle of the X-ray remained fixed while the detector was moved through a range of diffraction angles.

The surface analysis with an elemental quantification was investigated using X-ray photoelectron spectroscopy (XPS, PHI VersaProbe 3) that uses a monochromatized Al Kα radiation (1486 eV). All XPS spectra were calibrated to the C 1 s peak at a binding energy of 284.8 eV. CasaXPS software was used to carry out peak fitting with Shirley backgrounds.

Thermogravimetric and differential scanning calorimetry (TGA/DSC, Setaram Labsys Evo) were simultaneously used to obtain the heat flow and mass change on the precursor solution with the temperature range from 25 to 800 °C at a heating rate of 10 °C/min.

### Electrochemical measurement

Electrochemical measurement was conducted using a potentiostat (Model Interface 1010, Gamry) and three-electrode electrochemical cell including LaAlO_3_/FTO working electrode (geometric area of 0.502 cm^2^), carbon rod counter electrode^75,76^, and Ag/AgCl (KCl Sat.) reference electrode in the 30 mL of the electrolyte of 2 M KHCO_3_ (pH 8.3) and 4 M K_2_CO_3_/KHCO_3_ (1:7, pH 11). The measured potentials versus Ag/AgCl were converted to the potentials versus reversible hydrogen electrode (RHE) by the equation below:

5 $${E}_{{RHE}}={E}_{{Ag}/{AgCl}}+0.059\times {pH}+0.197$$

The electrochemical activity was evaluated using linear sweep voltammetry (LSV), sweeping from 1.2 V to 2.4 V vs. RHE at a scan rate of 50 mV/s. All curves are 95% iR-compensated. The EIS measurement was conducted between 20 kHz and 0.05 kHz at 2.7 V vs. RHE for all samples. Chronoamperometry assessed stability, applying a constant potential of 2.7 and 3.2 V vs. RHE for 3 h. H_2_O_2_ was collected during the chronoamperometry test at different times, followed by UV-vis absorbance measurement of the mixture solution of cobalt sulfate and the electrolyte, including the produced hydrogen peroxide. Before measuring the absorbance, the complexation time is required for 30 min. A calibration curve was measured with the same procedure but using commercial hydrogen peroxide [9]. H_2_O_2_ with higher concentration was quantified using the titration method with potassium permanganate and sulfuric acid, based on the following reaction:

$$2{{{{{{{\rm{MnO}}}}}}}_{4}}^{-}({{{{{\rm{aq}}}}}})\,+6{{{{{{\rm{H}}}}}}}^{+}({{{{{\rm{aq}}}}}})\,+\,5{{{{{{\rm{H}}}}}}}_{2}{{{{{{\rm{O}}}}}}}_{2}({{{{{\rm{aq}}}}}})\,\to 2{{{{{{\rm{Mn}}}}}}}^{2+}({{{{{\rm{aq}}}}}})\,+8{{{{{{\rm{H}}}}}}}_{2}{{{{{\rm{O}}}}}}\,({{{{{\rm{l}}}}}})\,+5{{{{{{\rm{O}}}}}}}_{2}({{{{{\rm{g}}}}}})$$

Selectivity was evaluated by the calculation of Faradaic efficiency, which is determined by the equation below:

6 $${FE}=\frac{n\times {N}_{{{H}_{2}O}_{2}}\times F}{I\times t}$$

where n, N_H2O2_, F, I, and t represent the number of electrons, the mole of H_2_O_2_ produced, faradaic constant, measured current, and time, respectively. The FEs at each potential were calculated by applying the absorbance measurement from its calibration curve (Supplementary Fig. 23) after the chronoamperometry test for 10 min, as shown in the Methods section. The inset picture in Supplementary Fig. 23 indicates the mixture of cobalt sulfate dye and the electrolyte, including the generated H_2_O_2_: the more greenish the solution looks, the more H_2_O_2_ is generated.

## Supplementary information

Supplementary Information

## Data Availability

The data that support the plots within this paper and other findings of this study are available from the corresponding authors on reasonable request.
