# Author Correction: Discovery of LaAlO_3_ as an efficient catalyst for two-electron water electrolysis towards hydrogen peroxide

**DOI:** 10.1038/s41467-022-35478-w

**Published:** 2022-12-12

**Authors:** Jihyun Baek, Qiu Jin, Nathan Scott Johnson, Yue Jiang, Rui Ning, Apurva Mehta, Samira Siahrostami, Xiaolin Zheng

**Affiliations:** 1 Department of Mechanical Engineering, Stanford University, Stanford, CA 94305 USA; 2 Department of Chemistry, University of Calgary, Calgary, AB T2N 1N4 Canada; 3 Stanford Synchrotron Radiation Lightsource, SLAC National Accelerator Laboratory, Menlo Park, CA 94025 USA; 4 Department of Materials Science and Engineering, Stanford University, Stanford, CA 94305 USA

**Keywords:** Electrocatalysis, Hydrogen energy, Electrocatalysis

Correction to: *Nature Communications* 10.1038/s41467-022-34884-4, published online 25 November 2022

The original version of this Article omitted an acknowledgement to the source of Supplementary Fig. 22. The following has been added to the main text and to the end of the caption to Supplementary Fig. 22: ‘reprinted (adapted) from ref. 68 Copyright 2021 American Chemical Society.’

The original version of this Article contained wrong reference for Ref. 33. The correct Ref. 33 is: Mavrikis, S. et al. Effective hydrogen peroxide production from electrochemical water oxidation. *ACS Energy Lett*. **6**, 2369–2377 (2021).

These have been corrected in the PDF and HTML versions of the Article.

## Supplementary information

Updated Supplementary Information

