# Supplementary material for: Discovery of LaAlO3 as an efficient catalyst for two-electron water electrolysis towards hydrogen peroxide
Source: Nat Commun. 2022 Nov 25;13:7256. doi: 10.1038/s41467-022-34884-4 (PMC9700689; doi:10.1038/s41467-022-34884-4)
Supplement: Supplementary file 1 — Supplementary Information [file 41467_2022_34884_MOESM1_ESM.pdf]

Supplementary Information

Discovery of $LaAlO_3$ as an Efficient Catalyst for Two-electron Water Electrolysis towards Hydrogen Peroxide

Jihyun Baek,[1,#] Qiu Jin,[2,#] Nathan Scott Johnson,[3] Yue Jiang,[1] Rui Ning,[4] Apurva Mehta,[3] Samira Siahrostami,[2,] and Xiaolin Zheng[1,*]*

[1]Department of Mechanical Engineering, Stanford University, Stanford, California 94305, United States

[2]Department of Chemistry, University of Calgary, 2500 University Drive NW, Calgary, Alberta, T2N 1N4, Canada

[3]Stanford Synchrotron Radiation Lightsource, SLAC National Accelerator

[4]Department of Materials Science and Engineering, Stanford University, Stanford, California 94305, United States

Laboratory, Menlo Park, California 94025, United States.

[#]These authors contributed equally to this work.

Supplementary Table 1 | Formation energies of promising perovskites for 2e-WOR under $pH_{electrolyte}$=8.

ABO_3 perovskites	Formation energy (eV/atom)
$LaAlO_3$	0.14
$LaCuO_3$	0.15
$PrAlO_3$	0.15
$EuNiO_3$	0.16
$LaNiO_3$	0.16
$PrCuO_3$	0.17
$SrTiO_3$	0.17
$GdAlO_3$	0.17
$SmCuO_3$	0.19
$SmNiO_3$	0.20

Supplementary Table 2 | Formation energies of promising perovskites for 2e-WOR under $pH_{electrolyte}$=11.

ABO_3 perovskites	Formation energy (eV/atom)
$LaAlO_3$	0.07
$SmCuO_3$	0.09
$SrTiO_3$	0.10
$GdAlO_3$	0.10
$YbCuO_3$	0.12
$YbTiO_3$	0.12
$SmNiO_3$	0.12
$GdCuO_3$	0.13
$YbNiO_3$	0.13
$LaGaO_3$	0.13
$PrCuO_3$	0.13
$NdCoO_3$	0.13
$YbAlO_3$	0.14
$TbNiO_3$	0.14
$LaCoO_3$	0.14
$DyAlO_3$	0.14
$SrCuO_3$	0.15
$PrAlO_3$	0.15
$SrNiO_3$	0.15
$SmCoO_3$	0.16
$TbCuO_3$	0.16
$BaTiO_3$	0.16
$LaZnO_3$	0.17
$EuFeO_3$	0.17
$EuCoO_3$	0.17
$YNiO_3$	0.18

$EuAgO_3$	0.19
$BaZrO_3$	0.19
$DyCuO_3$	0.19
$TaAgO_3$	0.19
$ErAlO_3$	0.19
$EuBeO_3$	0.19

Supplementary Table 3 | The calculated vibrational frequencies of the adsorbed species (O*, OH*, and OOH*) over $LaAlO_3$.

Structures with the adsorbates	Vibrational frequencies (cm^{-1})
O*	521.93
	128.94
	103.23
OH*	3798.98
	724.35
	516.88
	170.80
	148.74
	121.32
OOH*	3630.81
	1337.21
	918.74
	388.14
	266.29
	210.81
	116.92
	85.20
	59.58

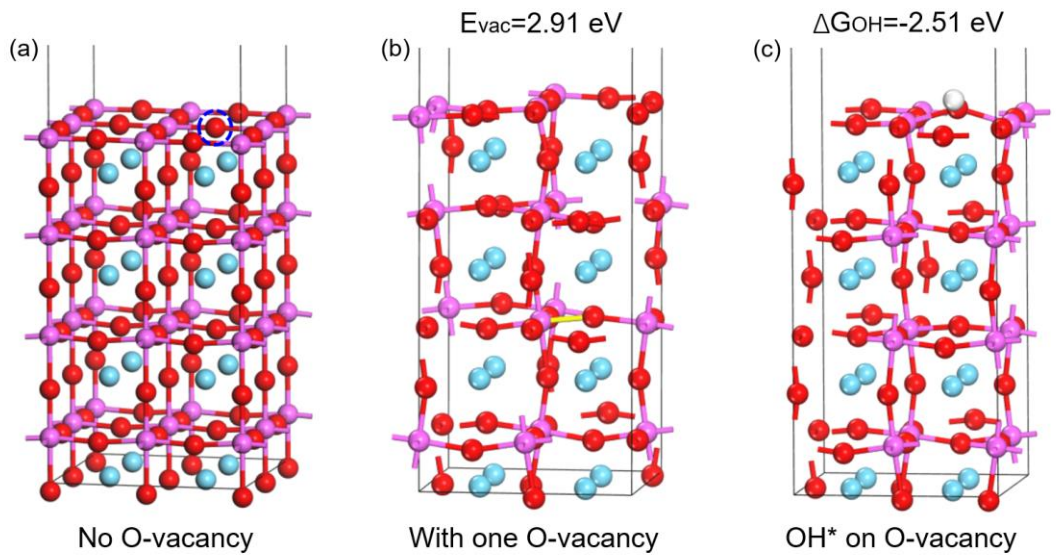

Supplementary Fig. 1 | Calculation of the O-vacancy formation over $LaAlO_3$. (a) $LaAlO_3$ with no O-vacancy. (b) $LaAlO_3$ with one O-vacancy. (c) OH* on O-vacancy. The O-vacancy position is marked by the blue dash circle in (a).

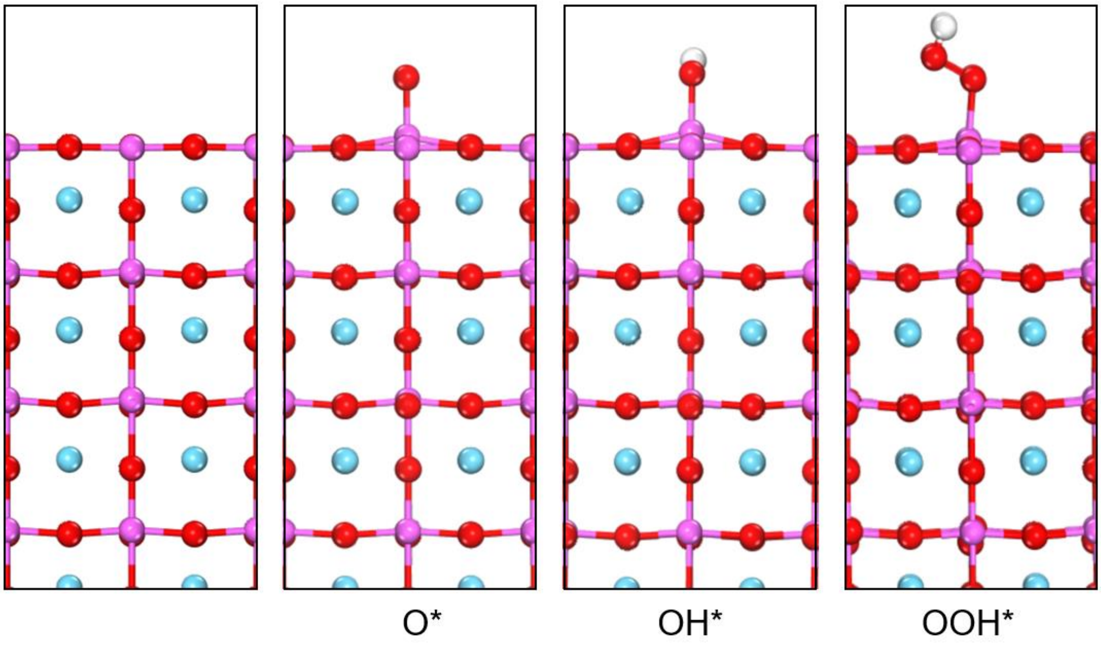

Supplementary Fig. 2 | Optimized DFT model structures of $LaAlO_3$ with O^*, OH^*, and OOH^* adsorption.

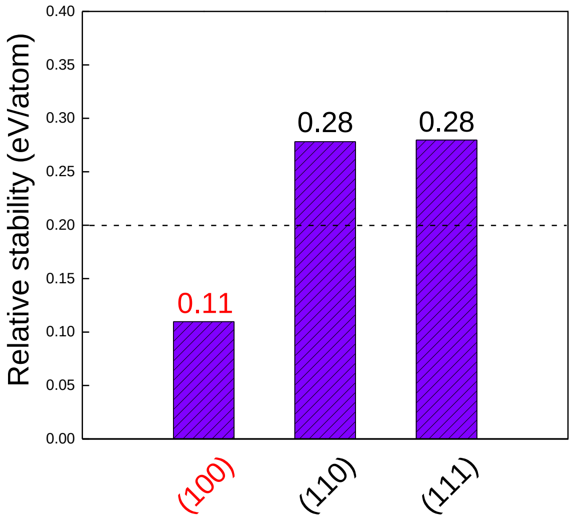

Supplementary Fig. 3 | Relative stability for (100), (110), and (111) facet $LaAlO_3$.

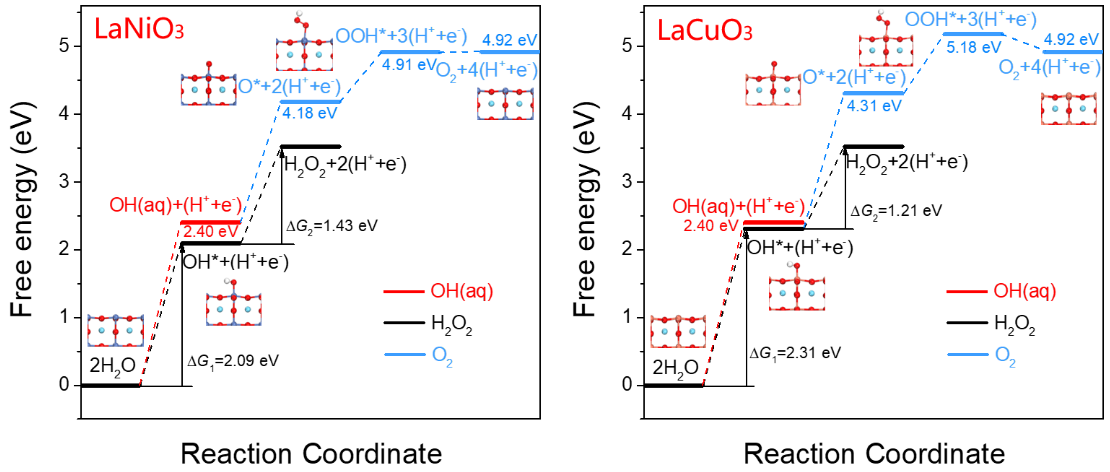

Supplementary Fig. 4 | Free energy diagram of all the three WORs over $LaNiO_3$ and $LaCuO_3$.

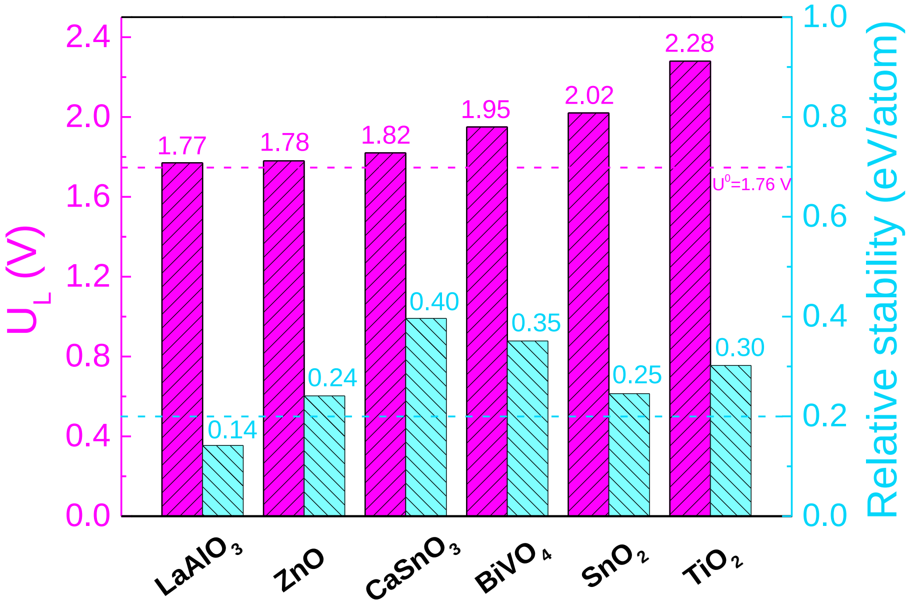

Supplementary Fig. 5 | Calculated limiting potentials (U_L) for the metal oxides (left axis) and the corresponding relative stability under 2e-WOR (electrolyte pH=8).

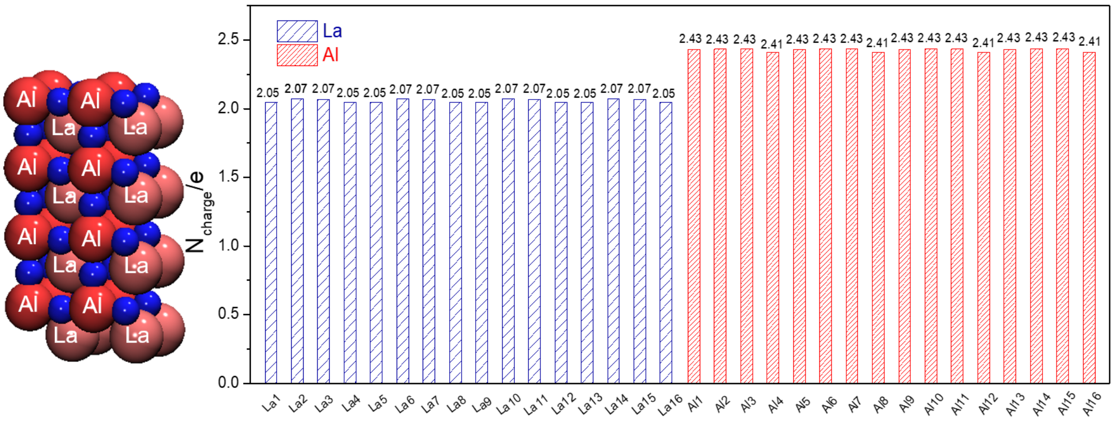

Supplementary Fig. 6 | Bader charge analysis of La and Al atoms in $LaAlO_3$.

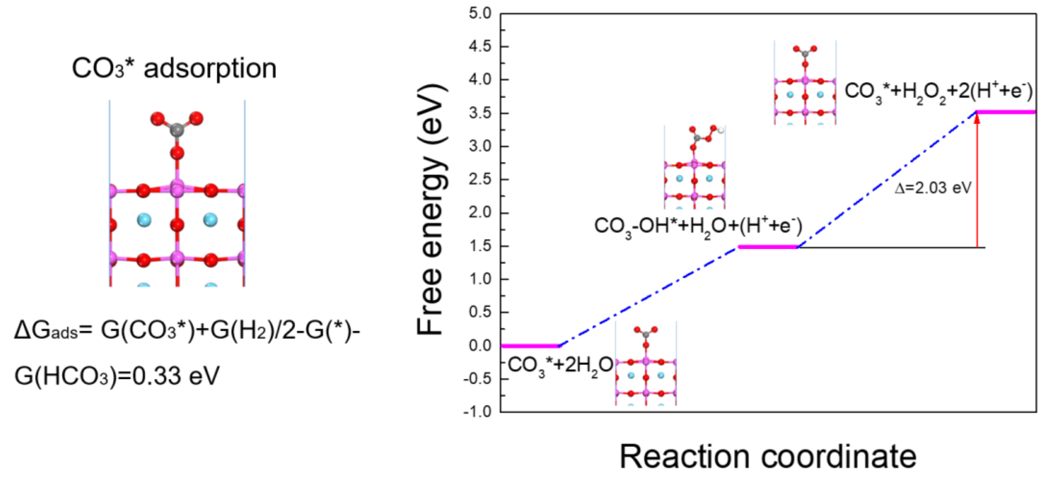

Supplementary Fig. 7 | The calculation of 2e-WOR via carbonate-mediated pathway on $LaAlO_3$.

Note:

The reaction pathway can be written as:

$$CO_3^*+H_2O \rightarrow CO_3\text{-}OH^*+(H^++e^-)$$

$$CO_3\text{-}OH^*+H_2O \rightarrow CO_3^*+H_2O_2+(H^++e\text{-})$$

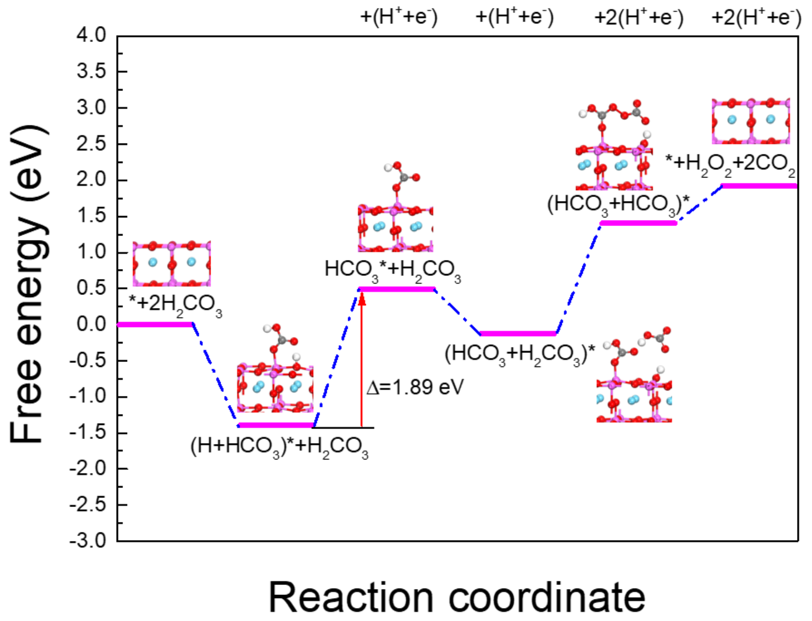

Supplementary Fig. 8 | Free energy diagram of H_2CO_3 oxidation on $LaAlO_3$.

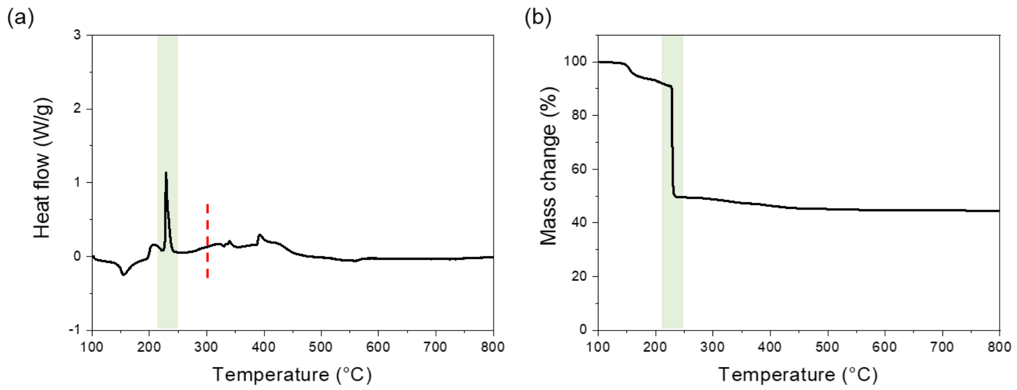

Supplementary Fig. 9 | TGA/DSC measurement for the precursor solution of $LaAlO_3$. The green region indicates the exothermic reaction (a), which corresponds to the rapid mass loss (b).

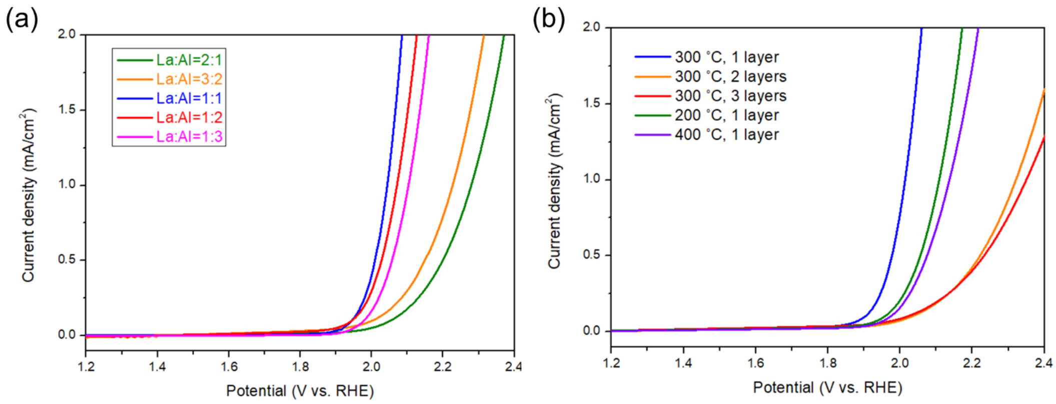

Supplementary Fig. 10 | Control experiment to optimize the activity of $LaAlO_3$ in 2M $KHCO_3$. (a) according to the precursor ratio between La and Al, (b) the number of layers or the synthesis temperature.

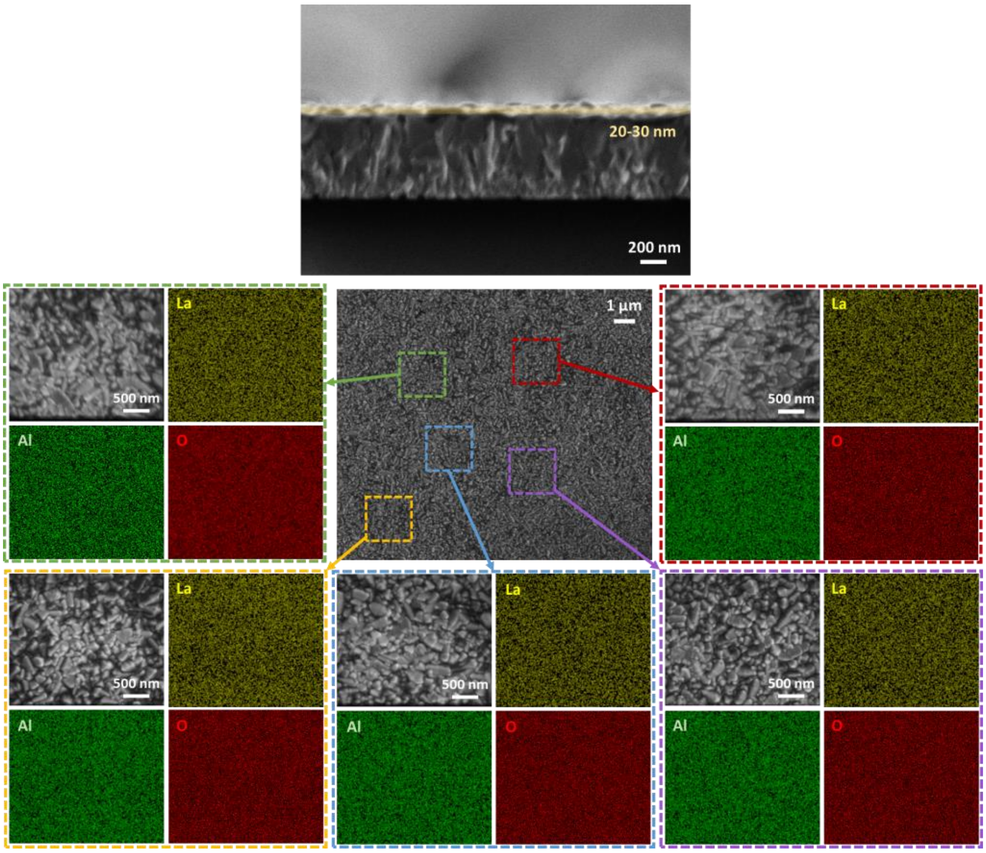

Supplementary Fig. 11 | SEM cross-view, top-view, and EDS mapping images of the synthesized $LaAlO_3$ on FTO/glass.

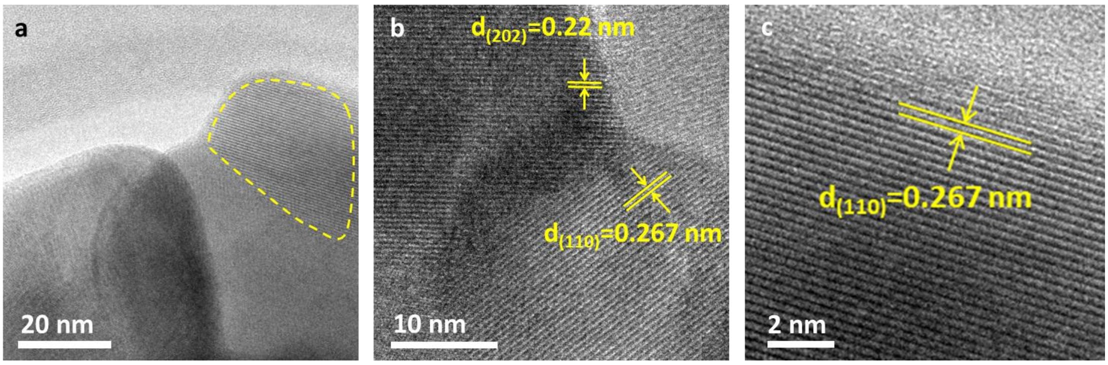

Supplementary Fig. 12 | HRTEM images of the synthesized $LaAlO_3$.

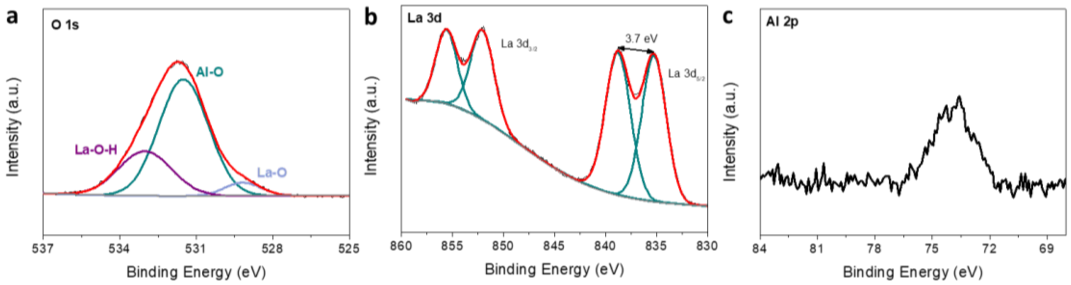

Supplementary Fig. 13 | XPS spectra of the $LaAlO_3$ thin film synthesized by the conventional sol-gel method.

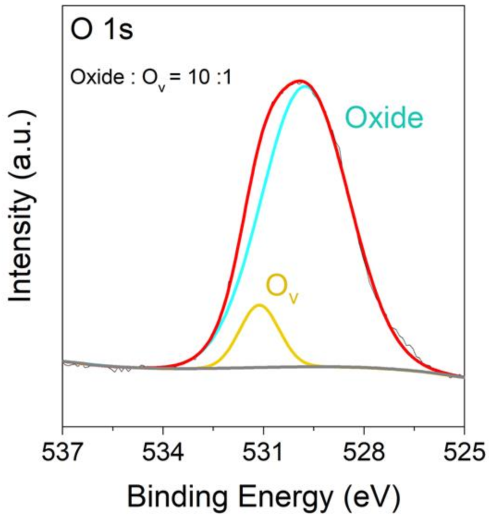

Supplementary Fig. 14 | XPS Surface analysis for the detailed O *1s* elemental region, indicating that the ratio of oxide to oxygen vacancy is roughly 10:1 based on its integrated area.

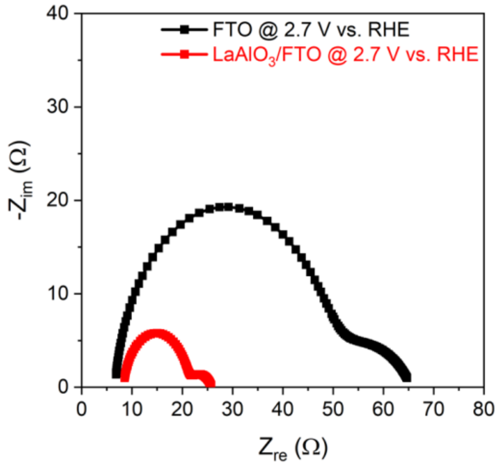

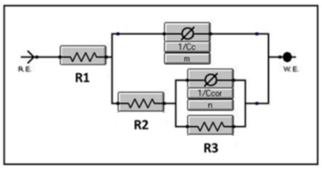

	R1	R2	R3
FTO	6.63	16.03	42.89
$LaAlO_3$/FTO	8.35	4.63	12.88

Supplementary Fig. 15 | EIS analysis measured at 2.7 V vs. RHE and fitted with a REAP2CPE equivalent circuit model in the Gamry instrument in which there were three types of resistances: resistance in the electrolyte solution (R1), charge transfer resistance inside bulk catalyst (R2), and charge transfer resistance at the interface between the catalyst and electrolyte (R3).

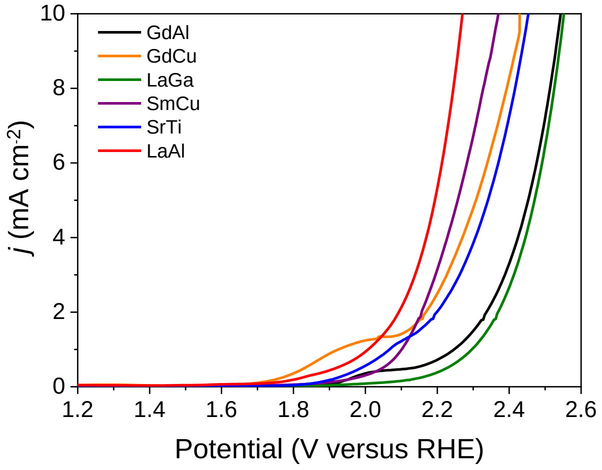

Supplementary Fig. 16 | LSV curves of different perovskite oxides, which are the most stable in the pH 11 condition from the calculation results.

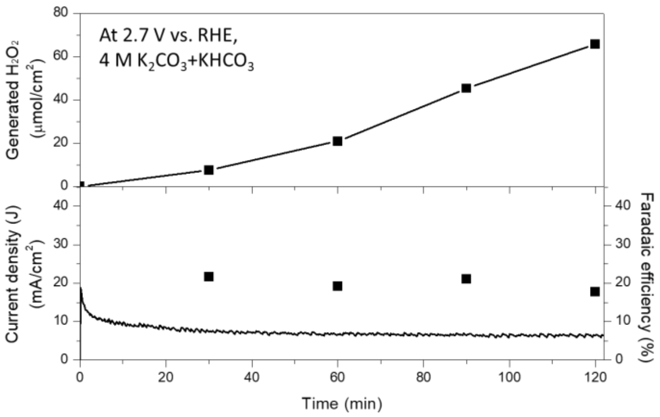

Supplementary Fig. 17 | Generated H_2O_2 amount, selectivity, and stability on FTO/glass substrate in 4 M K_2CO_3/$KHCO_3$ at 2.7 V vs. RHE.

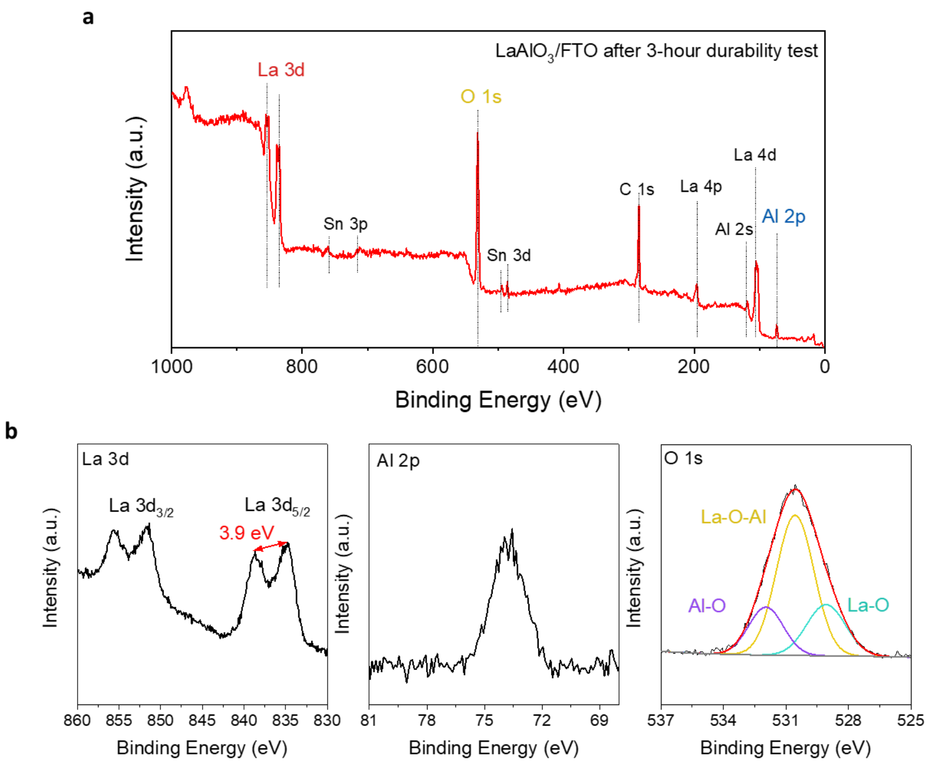

Supplementary Fig. 18 | Post-situ XPS spectra of $LaAlO_3$ on FTO substrate after 3 hours of the durability test.

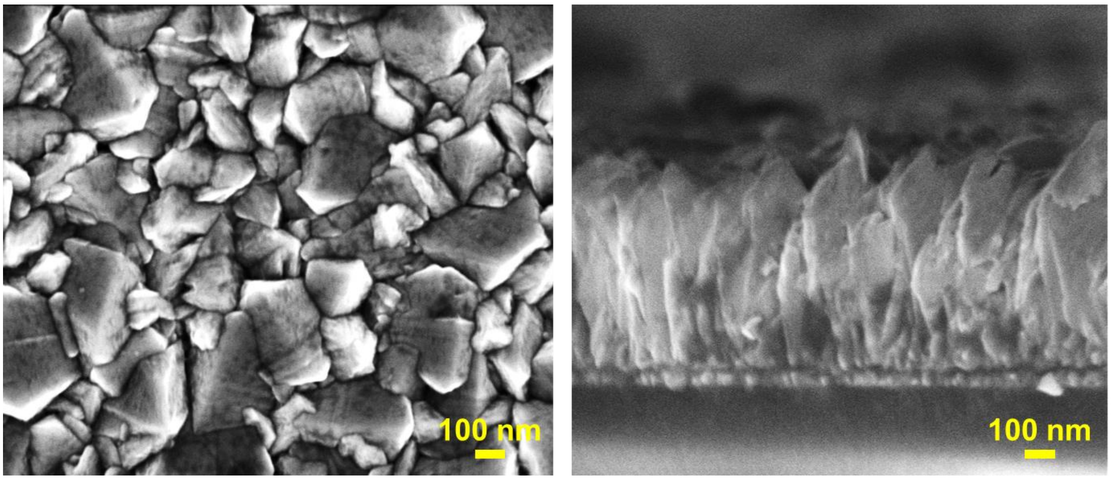

Supplementary Fig. 19 | Post-situ SEM top-view (left) and cross-view (right) images of $LaAlO_3$ on FTO substrate after 3 hours of the durability test.

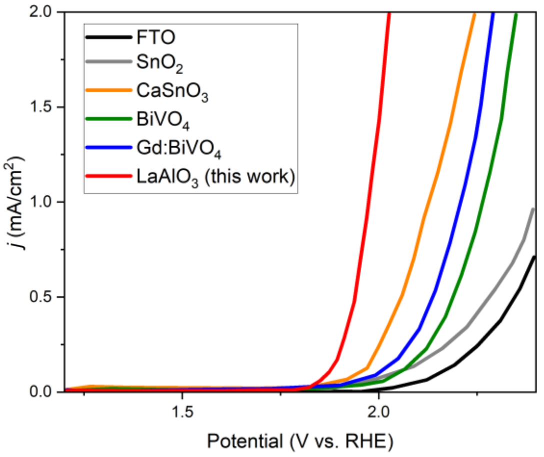

Supplementary Fig. 20 | LSV comparison of $LaAlO_3$ with different metal oxide catalysts in 2 M $KHCO_3$. Reproduced the LSV curves for FTO, SnO_2 and $CaSnO_3$ from ref. 19. Reproduced the LSV curves for $BiVO_4$ and Gd:$BiVO_4$ from ref. 25.

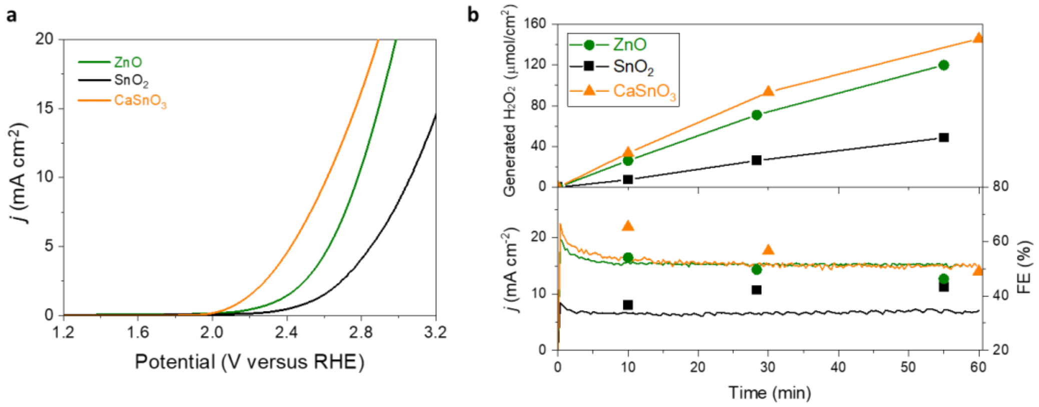

Supplementary Fig. 21 | Comparison of (a) LSV curves and (b) stability, Faradaic efficiency, and generated H_2O_2 amount of several state-of-the-art metal oxide thin films.

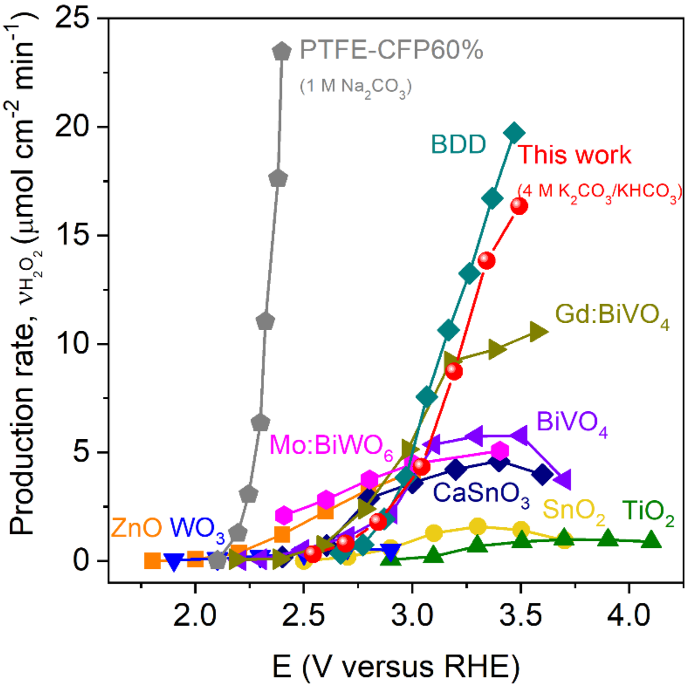

Supplementary Fig. 22 | Comparison of the production rates of the reported catalysts for H_2O_2 generation through 2e-WOR. This work was done in 4 M $K_2CO_3/KHCO_3$ (pH =11), and other works without any description were done in 2 M $KHCO_3$ (pH=8.3). Data were taken from ref. 9, 17, 18, 19, 20, 25, 29, 31, S[1], and S[2]. Reprinted (adapted) with permission from 68. Copyright 2021 American Chemical Society.

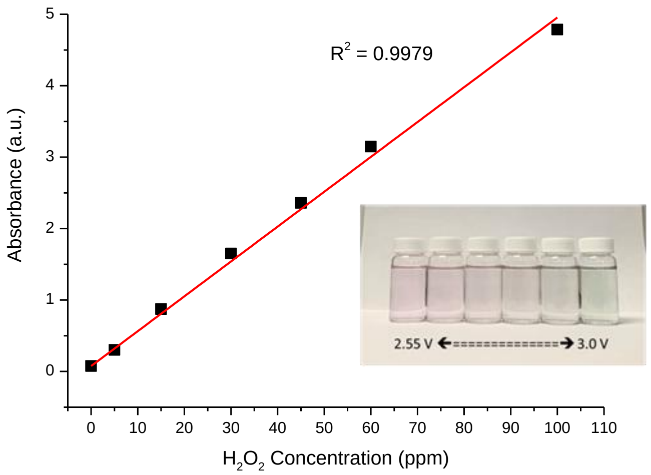

Supplementary Fig. 23 | Calibration curve from the UV-Vis measurement to calculate the produced H_2O_2 amount. The inset image shows the mixture of cobalt sulfate dye and the electrolyte, including the generated H_2O_2.

The coordinates of $LaAlO_3$-O*

La16Al16O49
1.0

7.6224999428	0.0000000000	0.0000000000
0.0000000000	7.6224999428	0.0000000000
0.0000000000	0.0000000000	28.3393993378

La	Al	O
16	16	49

Direct

0.249973997	0.249978006	0.000785000
0.250088006	0.250092000	0.138318002
0.249706998	0.249713004	0.276993990
0.253695011	0.253665000	0.416619986
0.750032008	0.249984995	0.000785000
0.749920011	0.250093013	0.138317004
0.750297010	0.249707997	0.276995003
0.746294022	0.253668010	0.416622013
0.249981999	0.750029981	0.000785000
0.250079006	0.749922991	0.138319999
0.249696001	0.750303984	0.276991993
0.253740996	0.746185005	0.416640013
0.750023007	0.750023007	0.000784000
0.749927998	0.749925971	0.138319001
0.750310004	0.750307024	0.276993006
0.746249974	0.746179998	0.416640013
0.999998987	0.999993980	0.062668003
0.000003000	0.000004000	0.202373996
0.000005000	0.999997020	0.340985000
0.999994993	0.999922991	0.473219991
0.500002980	0.999998987	0.062643997
0.500003994	0.000019000	0.202411994
0.500003994	0.999984026	0.340499014
0.499989986	0.999810994	0.474265009
0.999997020	0.499996990	0.062642001
0.000018000	0.500000000	0.202412993
0.000003000	0.500009000	0.340499997
0.999988019	0.499918997	0.474265009
0.499998987	0.499987990	0.062619999
0.500010014	0.499998003	0.202557996
0.500002980	0.500024021	0.341030002
0.499998987	0.499949992	0.486108005
0.249989003	0.999988973	0.058532000
0.250068009	0.999997020	0.197104007

0.250110000	0.000044000	0.335492015
0.249257997	0.999861002	0.474703997
0.999979973	0.249984995	0.058529001
0.999997020	0.250104010	0.197096005
0.000006000	0.250111997	0.335483015
0.999997973	0.249207005	0.474714011
0.000017000	0.000031000	0.988918006
0.000010000	0.000003000	0.128957003
0.999998987	0.000024000	0.267924011
0.000004000	0.000009000	0.406379998
0.750012994	0.999966025	0.058525000
0.749948025	0.000011000	0.197099999
0.749911010	0.000025000	0.335489988
0.750730991	0.999872983	0.474703997
0.499987990	0.249941006	0.058518000
0.500000000	0.250054985	0.197178006
0.500006020	0.249714002	0.335943997
0.499987006	0.245774999	0.474032015
0.499992996	0.000025000	0.988915980
0.500006974	0.000001000	0.128931999
0.500011027	0.000096000	0.268045008
0.499989986	0.999650002	0.406435013
0.249959007	0.499967009	0.058525998
0.250019997	0.500003994	0.197183996
0.249714002	0.500011981	0.335911006
0.245901003	0.499888003	0.474115998
0.999972999	0.749997020	0.058525998
0.000000000	0.749957979	0.197100997
0.000009000	0.749925971	0.335494012
0.999994993	0.750657976	0.474707007
0.999988019	0.500027001	0.988916993
0.000020000	0.500003994	0.128932998
0.000010000	0.500010014	0.268043011
0.999970973	0.499936014	0.406424999
0.750041008	0.499965012	0.058527999
0.749947011	0.500018001	0.197183996
0.750306010	0.500015020	0.335905999
0.754077971	0.499882996	0.474132001
0.499996006	0.750042021	0.058525000
0.500000000	0.749989986	0.197194993
0.500002980	0.750347972	0.335871994
0.499985993	0.753901005	0.474229008
0.500002980	0.500048995	0.988928974
0.500000000	0.500029027	0.128909007

0.499998003	0.499942005	0.268222004
0.500011981	0.500194013	0.406673014
0.499770999	0.497965008	0.549517989

The coordinates of $LaAlO_3$-OH*

La16Al16O49H1
1.0

7.6224999428	0.0000000000	0.0000000000
0.0000000000	7.6224999428	0.0000000000
0.0000000000	0.0000000000	28.3393993378

La	Al	O	H
16	16	49	1

Direct

0.249968007	0.250095993	0.000081000
0.250092000	0.250167996	0.137803003
0.249204993	0.249357000	0.276771992
0.255710006	0.256220996	0.416761994
0.750042975	0.249939993	0.000083000
0.749844015	0.250252008	0.137783006
0.750603974	0.249073997	0.276740015
0.743326008	0.255914986	0.416520000
0.249947995	0.750020981	0.000078000
0.250173002	0.749934971	0.137815997
0.249087006	0.750533998	0.276751995
0.255742013	0.743336976	0.416779995
0.750060022	0.750167012	0.000081000
0.749764979	0.749859989	0.137796998
0.750720978	0.750823021	0.276717991
0.743336976	0.743685007	0.416503996
0.000015000	0.000067000	0.061965000
0.999936998	0.000054000	0.201896995
0.999972999	0.999929011	0.341275007
0.999395013	0.999769986	0.472921997
0.499967992	0.000031000	0.061918002
0.499981999	0.000081000	0.202015996
0.499749988	0.999920011	0.340382993
0.499574006	0.999701023	0.474476993
0.999935985	0.500078976	0.061919000
0.000017000	0.500050008	0.202030003
0.999828994	0.499949008	0.340407997
0.998610020	0.499790013	0.474440992
0.500011027	0.500106990	0.061889000
0.499844015	0.500010014	0.202207997

0.499761999	0.499917001	0.340086997
0.502054989	0.499855012	0.490859985
0.249991000	0.000001000	0.057619002
0.250032008	0.000135000	0.196813002
0.249906003	0.999909997	0.335240990
0.248932004	0.999697030	0.475542992
0.999994993	0.250066996	0.057679001
0.999827027	0.250138015	0.196669996
0.999898016	0.249945998	0.335388988
0.999216020	0.249252006	0.475217015
0.999451995	0.000169000	0.988188028
0.000635000	0.000142000	0.128297001
0.999017000	0.999836028	0.267538995
0.000797000	0.000017000	0.406549007
0.749988973	0.000043000	0.057856001
0.749899983	0.000044000	0.196404994
0.749825001	0.999894977	0.335633010
0.749971986	0.999682009	0.474911988
0.499920994	0.250072002	0.057728000
0.500097990	0.250185996	0.196567997
0.499935001	0.249197006	0.335606009
0.499496996	0.246326998	0.474900007
0.500418007	0.000295000	0.988192976
0.499379009	0.999743998	0.128272995
0.500864029	0.000629000	0.267612010
0.498789012	0.999435008	0.406551987
0.249943003	0.500025988	0.057670001
0.250021011	0.500014007	0.196593001
0.249085993	0.499967009	0.335734993
0.245511994	0.499711990	0.475566000
0.000048000	0.750097990	0.057684001
0.999934018	0.749978006	0.196628004
0.999899983	0.749880016	0.335442990
0.999234021	0.750253975	0.475142986
0.999782979	0.500132978	0.988201022
0.999943972	0.499976009	0.128273994
0.000702000	0.500200987	0.267632991
0.998609006	0.499736011	0.406598002
0.749988019	0.500154018	0.057734001
0.749827027	0.500135005	0.196789995
0.750512004	0.499978989	0.335281014
0.752417982	0.499752998	0.475439996
0.499938995	0.750059009	0.057650000
0.499991000	0.749978006	0.196811005

0.499974996	0.750639975	0.335355014
0.499581993	0.753122985	0.475021005
0.500025988	0.500047028	0.988205016
0.499945015	0.500500977	0.128224999
0.498941988	0.499372989	0.267908990
0.500145018	0.500200987	0.405366004
0.504113019	0.499101996	0.553883016
0.387843013	0.493966013	0.567759991

The coordinates of $LaAlO_3$-OOH*

La16Al16O50H1
1.0

7.6224999428	0.0000000000	0.0000000000
0.0000000000	7.6224999428	0.0000000000
0.0000000000	0.0000000000	28.3393993378

La	Al	O	H
16	16	50	1

Direct

0.250741988	0.261633009	0.999921024
0.250730008	0.246659994	0.137975007
0.250719994	0.249079004	0.277328014
0.254496992	0.251228988	0.417524010
0.750441015	0.234402999	0.999817014
0.750738025	0.250173002	0.137860000
0.751021028	0.246913999	0.277465999
0.747116029	0.251031995	0.417540014
0.250319004	0.734205008	0.999819994
0.250909001	0.749800980	0.137923002
0.250620008	0.747144997	0.277282000
0.254619986	0.744287014	0.417652994
0.750750005	0.761943996	0.999893010
0.750568986	0.746545970	0.137939006
0.751085997	0.749603987	0.277386993
0.747107983	0.744381011	0.417751014
0.003251000	0.999141991	0.062532999
0.001440000	0.999136984	0.202681005
0.000024000	0.998506010	0.341704011
0.002659000	0.997860014	0.473803014
0.497886002	0.999019980	0.062503003
0.500046015	0.999161005	0.202737004
0.501686990	0.998601019	0.341316998
0.499312013	0.997946978	0.474783003
0.997902989	0.499038011	0.062481999

0.999827981	0.499128997	0.202711999
0.001751000	0.498735994	0.341264993
0.999854982	0.497653008	0.474489987
0.503147006	0.499215007	0.062465001
0.501608014	0.499139011	0.202837005
0.499960989	0.498670995	0.341780990
0.502278984	0.497310013	0.484162003
0.250526011	0.992148995	0.051073000
0.250824004	0.007918000	0.202122003
0.250941008	0.993201017	0.332839012
0.250640988	0.998242021	0.477537006
0.008180000	0.249318004	0.057641000
0.993013024	0.249362007	0.196888000
0.006896000	0.248835996	0.335772008
0.000630000	0.247263998	0.475398004
0.968656003	0.999538004	0.987933993
0.023745000	0.999395013	0.128313005
0.984167993	0.998349011	0.267995000
0.011670000	0.998467982	0.407052994
0.750509977	0.007351000	0.064377002
0.750626981	0.992615998	0.192405999
0.750706971	0.005690000	0.339619011
0.751381993	0.997411013	0.473937988
0.492639989	0.249346003	0.057912000
0.508324981	0.249321997	0.196509004
0.494529992	0.248637006	0.336483985
0.501151979	0.245268002	0.474851012
0.532513976	0.000481000	0.987881005
0.477797002	0.997877002	0.128279001
0.517234981	0.999894977	0.268148005
0.491156995	0.997363985	0.407097012
0.250506997	0.507517993	0.064291999
0.250735015	0.492630005	0.192613006
0.250699013	0.505533993	0.339607000
0.247639999	0.497305006	0.473852992
0.992623985	0.749391973	0.057813998
0.008316000	0.749246001	0.196657002
0.994647026	0.748714030	0.335860014
0.001462000	0.748225987	0.475373000
0.032230001	0.500036001	0.987958014
0.978233993	0.498400003	0.128291994
0.016264999	0.499191999	0.268110007
0.992497027	0.498252004	0.406993002
0.750522017	0.492228001	0.051169999

0.750675976	0.508019984	0.201949999
0.750873983	0.493155986	0.333510011
0.754315972	0.497779995	0.476561993
0.508156002	0.749382973	0.057436001
0.493097007	0.749307990	0.197123006
0.506718993	0.748916984	0.335920990
0.500504971	0.750675023	0.475537986
0.468614012	0.499060988	0.987945974
0.523042023	0.500158012	0.128259003
0.485022992	0.497572988	0.268180996
0.509299994	0.499329001	0.407561988
0.541014016	0.360814989	0.575384021
0.491600990	0.517569005	0.551477015
0.535274982	0.401735008	0.608151019

The coordinates of $LaAlO_3$-CO_3*

La16Al16O51C1
1.0

7.6224999428	0.0000000000	0.0000000000
0.0000000000	7.6224999428	0.0000000000
0.0000000000	0.0000000000	28.3393993378

La	Al	O	C
16	16	51	1

Direct

0.249972999	0.249927998	0.000787000
0.250063986	0.249988005	0.138248995
0.249594003	0.249797001	0.276814997
0.254009992	0.254449993	0.416269004
0.750037014	0.249919996	0.000787000
0.749943018	0.249978006	0.138248995
0.750410974	0.249806002	0.276814014
0.745989025	0.254444987	0.416258991
0.250016004	0.749966025	0.000789000
0.250081986	0.749908984	0.138238996
0.249816000	0.750256002	0.276829004
0.253987014	0.746722996	0.416110009
0.749993980	0.749971986	0.000789000
0.749925017	0.749917984	0.138239995
0.750186026	0.750254989	0.276831001
0.746016026	0.746725023	0.416110009
0.999997020	0.999930978	0.062668003
0.000001000	0.999987006	0.202399001
0.000000000	0.000181000	0.340921998

0.000006000	0.000621000	0.473334998
0.499998987	0.999921978	0.062657997
0.500006974	0.999881029	0.202393994
0.500002980	0.000209000	0.340382993
0.500002980	0.000660000	0.474038988
0.000005000	0.499922007	0.062659003
0.000006000	0.499985993	0.202373996
0.000002000	0.500132024	0.340492994
0.999975026	0.500707984	0.474411994
0.499996006	0.499922007	0.062657997
0.500007987	0.500078976	0.202467993
0.499998003	0.500065982	0.340838999
0.499972999	0.500877023	0.484757990
0.249988005	0.999894023	0.058653001
0.250003010	0.000008000	0.197238997
0.250066996	0.000026000	0.335491002
0.249566004	0.000865000	0.474754006
0.999988973	0.249892995	0.058669001
0.000011000	0.249987006	0.197228998
0.000017000	0.250303000	0.335586995
0.000021000	0.250283986	0.474774987
0.999974012	0.999909997	0.989216983
0.000005000	0.999983013	0.129041001
0.999993026	0.000201000	0.268020004
0.000026000	0.000330000	0.406408012
0.750002980	0.999890983	0.058653001
0.750010014	0.999994993	0.197237000
0.749933004	0.000036000	0.335505992
0.750428021	0.000880000	0.474747986
0.499992996	0.249862000	0.058651999
0.500002027	0.250037998	0.197284997
0.499983996	0.250001013	0.335525990
0.499987006	0.247769997	0.474559993
0.499985993	0.999808013	0.989233971
0.500024021	0.999983013	0.129032001
0.500018001	0.999131024	0.268070012
0.499994993	0.001443000	0.406264007
0.249965996	0.499890000	0.058685001
0.250041008	0.499978989	0.197255000
0.249798998	0.500074983	0.335969001
0.247442007	0.500733972	0.474465996
0.999989986	0.749916971	0.058658000
0.999993980	0.749985993	0.197233006
0.999974012	0.749993026	0.335579991

0.999993026	0.751101017	0.474812001
0.999989986	0.499832988	0.989247024
0.000045000	0.499944001	0.129041001
0.999971986	0.499873996	0.268099010
0.000014000	0.500545979	0.406491995
0.750033975	0.499891996	0.058687001
0.749975979	0.499976009	0.197252005
0.750205994	0.500036001	0.335979015
0.752548993	0.500715017	0.474462986
0.499992996	0.749939978	0.058695000
0.500012994	0.749942005	0.197175995
0.500003994	0.750151992	0.336084992
0.500010014	0.753835976	0.473933011
0.499976993	0.499945998	0.989273012
0.500002027	0.499938011	0.129034996
0.500028014	0.500899017	0.268168986
0.499992013	0.499581009	0.406664014
0.500128984	0.505639970	0.549965978
0.641857028	0.505928993	0.618752003
0.354954004	0.494015008	0.618192017
0.498955011	0.501555979	0.595627010

The coordinates of $LaAlO_3$-(CO_3-OH)*

La16Al16O52C1H1
1.0

7.6224999428	0.0000000000	0.0000000000
0.0000000000	7.6224999428	0.0000000000
0.0000000000	0.0000000000	28.3393993378

La	Al	O	C	H
16	16	52	1	1

Direct

0.229252994	0.251381993	0.000457000
0.244813994	0.251111001	0.138374999
0.241182998	0.250669003	0.277776986
0.248353004	0.257615000	0.417771995
0.756902993	0.250719994	0.000538000
0.741405010	0.251345009	0.138421997
0.744921029	0.250456989	0.277673990
0.737163007	0.257584006	0.417901009
0.256704003	0.750832975	0.000555000
0.241679996	0.751051009	0.138410002
0.243388996	0.751782000	0.277693003
0.248237997	0.746487975	0.417661011

0.729179978	0.751345992	0.000465000
0.744494021	0.750891984	0.138393998
0.742393017	0.752008021	0.277707994
0.737236023	0.746605992	0.417760998
0.994624972	0.998667002	0.063336998
0.994198978	0.000443000	0.203235000
0.993562996	0.002232000	0.342759997
0.992981017	0.000072000	0.474682987
0.494415998	0.003765000	0.063272998
0.494186014	0.002011000	0.203384995
0.493683010	0.000611000	0.341955006
0.492630005	0.003137000	0.476128995
0.994377971	0.503766000	0.063285001
0.994302988	0.501980007	0.203345001
0.993501008	0.500468016	0.341915011
0.992475986	0.503275990	0.475973994
0.494583011	0.498613000	0.063282996
0.494105011	0.500463009	0.203529000
0.493597001	0.502215028	0.341701001
0.492953002	0.501504004	0.490256995
0.244914994	0.990163982	0.058543999
0.244546995	0.011734000	0.198004007
0.243784994	0.994113028	0.336578995
0.242566004	0.002234000	0.476785988
0.006670000	0.251138002	0.065856002
0.985529006	0.251291007	0.192973003
0.002145000	0.251368999	0.340995014
0.992232025	0.251358986	0.474541992
0.994711995	0.034357000	0.989337981
0.995297015	0.977344990	0.129299000
0.992403984	0.018972000	0.268745989
0.994086027	0.988334000	0.408062011
0.744948983	0.012294000	0.059007999
0.744288981	0.991038978	0.197363004
0.743728995	0.008731000	0.337172002
0.743187010	0.001217000	0.476516008
0.485085994	0.251361012	0.051890001
0.506438971	0.251314014	0.203484997
0.487369001	0.250656992	0.333380014
0.493544012	0.249483004	0.478868008
0.496203989	0.968361020	0.989395022
0.493146986	0.025246000	0.129212007
0.494735986	0.982926011	0.268853992
0.492181987	0.012748000	0.408118993

0.244837999	0.512390971	0.059064001
0.244473994	0.491046995	0.197272003
0.243016005	0.508841991	0.337442011
0.240250006	0.501425028	0.475822985
0.985069990	0.751330018	0.051872000
0.006355000	0.751160979	0.203435004
0.987383008	0.751362979	0.333361000
0.993494987	0.751766026	0.478859991
0.996290028	0.468178004	0.989355981
0.992874980	0.525213003	0.129221007
0.995504022	0.482971996	0.268821001
0.990558982	0.512894988	0.407985985
0.744947016	0.490200996	0.058451999
0.744418979	0.511807024	0.198146001
0.744315982	0.494452000	0.336237013
0.744850993	0.502503991	0.476960003
0.506686985	0.751187027	0.065857001
0.485469013	0.751147985	0.193027005
0.501888990	0.752022028	0.341109991
0.492558002	0.754464984	0.475091994
0.494406998	0.534325004	0.989369988
0.495541990	0.477389008	0.129242003
0.491569012	0.519243002	0.269057989
0.494843006	0.490036994	0.407040000
0.471195996	0.512760997	0.554247022
0.721813023	0.488721997	0.592247009
0.459307998	0.488178998	0.634203970
0.801074982	0.474862993	0.639634013
0.534704030	0.496035010	0.596279979
0.924284995	0.480590999	0.629541993

The coordinates of $LaAlO_3$-(H+HCO_3)*

La16Al16O51C1H2
1.0

7.6224999428	0.0000000000	0.0000000000
0.0000000000	7.6224999428	0.0000000000
0.0000000000	0.0000000000	28.3393993378

La	Al	O	C	H
16	16	51	1	2

Direct

0.227163002	0.252539009	0.003417000
0.241745993	0.249140993	0.141045004
0.239453003	0.251601011	0.280961990

0.242682993	0.247380003	0.419701010
0.753934026	0.247688994	0.003352000
0.739028990	0.250665992	0.141489998
0.740669012	0.249162003	0.280111998
0.735516012	0.248714998	0.421739012
0.252344012	0.747651994	0.003441000
0.238052994	0.751164019	0.141058996
0.239363000	0.748717010	0.280800015
0.242189005	0.753126979	0.419501990
0.725549996	0.752548993	0.003352000
0.740536988	0.749733984	0.141475007
0.738286972	0.751013994	0.279949993
0.734164000	0.752817988	0.421296000
0.990364015	0.997757018	0.065936998
0.990281999	0.999179006	0.206021994
0.988339007	0.001197000	0.344267994
0.991087973	0.998408020	0.476732999
0.489367008	0.002495000	0.065939002
0.489363015	0.001215000	0.206015006
0.490451992	0.999031007	0.344029993
0.487120986	0.001211000	0.477824986
0.988810003	0.502565026	0.066013999
0.990895987	0.501124024	0.205959007
0.987010002	0.499112010	0.344862014
0.995702982	0.501982987	0.475964993
0.490851015	0.497669995	0.065995999
0.488584012	0.499053001	0.206056997
0.491310000	0.501088977	0.345853001
0.473398000	0.499787986	0.486337006
0.239916995	0.999614000	0.060130998
0.239905000	0.999719977	0.201552004
0.239706993	0.000515000	0.338380009
0.239102006	0.999221027	0.478053987
0.990101993	0.250214994	0.067814998
0.990487993	0.250151008	0.196382001
0.988456011	0.250696003	0.341533005
0.991859972	0.250099003	0.475667000
0.985274017	0.031688001	0.991711974
0.994701982	0.979086995	0.131840006
0.984385014	0.013475000	0.271450996
0.992547989	0.993171990	0.409631997
0.739879012	0.000655000	0.062371001
0.739829004	0.000556000	0.199063003
0.739229023	0.999309003	0.340469003

0.739497006	0.000599000	0.477914006
0.489573002	0.250059009	0.055002999
0.489113003	0.250106007	0.204839006
0.490193993	0.250762999	0.337464005
0.487668991	0.248301998	0.477914989
0.494448006	0.968578994	0.991696000
0.485096008	0.021091999	0.131860003
0.495067000	0.987164021	0.271562010
0.486535013	0.005480000	0.409808993
0.239809006	0.500665009	0.063936003
0.239832997	0.500571012	0.196420997
0.239107996	0.499639004	0.345670015
0.234905005	0.500813007	0.472016990
0.989235997	0.750145018	0.054981001
0.989790022	0.750113010	0.204918995
0.989791989	0.749423027	0.336618990
0.989642978	0.749629021	0.477748990
0.998239994	0.468928009	0.991739988
0.978037000	0.521308005	0.131895006
0.007415000	0.487194002	0.271375000
0.958913028	0.506329000	0.410488993
0.739875019	0.499651015	0.058717001
0.739818990	0.499668986	0.204091996
0.738997996	0.500738978	0.334031999
0.747897983	0.495238006	0.490480989
0.490862012	0.750100970	0.067824997
0.490386009	0.750108004	0.196507007
0.489396989	0.749472022	0.342070997
0.489295989	0.751899004	0.476172000
0.481323004	0.531113982	0.991819978
0.501551986	0.479245991	0.131856993
0.471320003	0.512803018	0.271414012
0.515505016	0.495148987	0.411947995
0.444319010	0.504913986	0.552002013
0.718595028	0.459553987	0.582040012
0.485009998	0.481305003	0.630267024
0.556757987	0.481236011	0.585693002
0.359133005	0.498434991	0.625678003
0.746964991	0.476624012	0.526443005

The coordinates of $LaAlO_3$-HCO_3*

La16Al16O51C1H1
1.0

7.6224999428	0.0000000000	0.0000000000
0.0000000000	7.6224999428	0.0000000000
0.0000000000	0.0000000000	28.3393993378

La Al O C H

16 16 51 1 1

Direct

0.231362998	0.250122994	0.999741971
0.246988997	0.250257999	0.137626007
0.242709994	0.249357998	0.276867002
0.249198005	0.256343991	0.417050987
0.759742022	0.250138015	0.999688983
0.743376017	0.250176996	0.137623996
0.746454000	0.249342993	0.276897997
0.739184976	0.256074995	0.416729003
0.259644002	0.750185013	0.999684989
0.243686005	0.749917984	0.137610003
0.245297000	0.750608981	0.276820004
0.249230996	0.744789004	0.416933000
0.731402993	0.750159025	0.999723971
0.746653020	0.749957025	0.137606993
0.743995011	0.750639975	0.276816010
0.739359021	0.745163977	0.416588992
0.994907022	0.997434020	0.062449999
0.994732022	0.999311984	0.202354997
0.994601011	0.000847000	0.341769993
0.994130015	0.998614013	0.473818004
0.494928986	0.002828000	0.062410999
0.494731009	0.000869000	0.202447996
0.494300008	0.999213994	0.340970010
0.494417995	0.001658000	0.474851996
0.994922996	0.502852976	0.062419999
0.994684994	0.500778973	0.202460006
0.994547009	0.499058008	0.341048986
0.993340015	0.501877010	0.475706995
0.494899005	0.497458011	0.062392000
0.494767994	0.499302000	0.202620000
0.494266987	0.500945985	0.340802997
0.495400012	0.499767005	0.488584995
0.244793996	0.004424000	0.057812002
0.244801998	0.997174978	0.196578994
0.244461000	0.000748000	0.335714012
0.243956998	0.000329000	0.475639999
0.990216017	0.250097007	0.064995997
0.997429013	0.250113010	0.191834003

0.993376970	0.250000000	0.340315998
0.994096994	0.249886006	0.473538011
0.994656980	0.033975001	0.988218009
0.994558990	0.975295007	0.128331006
0.994983017	0.018998999	0.267791986
0.994602025	0.985966980	0.407090008
0.744808018	0.995728016	0.057746999
0.744669974	0.002997000	0.196713001
0.744479001	0.999090016	0.335741013
0.744642973	0.000204000	0.475445002
0.498726010	0.250135005	0.050719000
0.491252005	0.250117987	0.202694997
0.495440006	0.249406993	0.332130015
0.494486004	0.247519001	0.477726996
0.494491011	0.966363013	0.988252997
0.495014995	0.024675000	0.128304005
0.494724005	0.981023014	0.267856002
0.494392991	0.012166000	0.406964988
0.244721994	0.495761991	0.057760000
0.244779006	0.503058016	0.196766004
0.243797004	0.499401987	0.335640013
0.241648003	0.500469983	0.475753009
0.998700976	0.750150025	0.050684001
0.991357028	0.750027001	0.202735007
0.995132029	0.749895990	0.332170993
0.994057000	0.750546992	0.478199989
0.994445980	0.466325998	0.988256991
0.994964004	0.524873018	0.128325000
0.994346976	0.480717003	0.267877996
0.995407999	0.512266994	0.407135010
0.744823992	0.504576027	0.057822000
0.744646013	0.497177005	0.196622998
0.744980991	0.500926018	0.336012989
0.745917976	0.500625014	0.474682987
0.490309000	0.750141978	0.064984001
0.497411013	0.749969006	0.191906005
0.493391007	0.750579000	0.340261996
0.494529009	0.753259003	0.473798990
0.494480014	0.533883989	0.988269985
0.494531989	0.475488007	0.128280997
0.495166004	0.519021988	0.268081993
0.493149996	0.488182008	0.406152010
0.478902012	0.509195983	0.553013980
0.745069981	0.485372990	0.589929998

0.494524002	0.499796003	0.631969988
0.583458006	0.497366995	0.589388013
0.370177001	0.508230984	0.623842001

The coordinates of $LaAlO_3$-(HCO_3+H_2CO_3)*

La16Al16O54C2H3
1.0

7.6224999428	0.0000000000	0.0000000000
0.0000000000	7.6224999428	0.0000000000
0.0000000000	0.0000000000	28.3393993378

La	Al	O	C	H
16	16	54	2	3

Direct

0.225796998	0.248656005	0.999314010
0.241595998	0.248685002	0.136866003
0.236172006	0.248083994	0.276461989
0.248317003	0.253991991	0.416476011
0.754374027	0.248693004	0.999307990
0.737169027	0.248747006	0.136892006
0.741292000	0.248072997	0.276436001
0.728955984	0.253912002	0.416014999
0.254289001	0.748773992	0.999216974
0.238428995	0.748485029	0.137053996
0.239310995	0.748979986	0.275604993
0.248328999	0.744499981	0.416191012
0.725769997	0.748705029	0.999280989
0.740552008	0.748520017	0.137049004
0.738133013	0.749065995	0.275483012
0.724656999	0.744500995	0.414743990
0.989427984	0.995763004	0.062120002
0.989120007	0.998724997	0.201745003
0.988903999	0.997304976	0.341710001
0.987874985	0.005276000	0.471964002
0.489371002	0.001278000	0.061983000
0.489161998	0.999701977	0.201967001
0.488427013	0.997891009	0.339783996
0.489302009	0.001227000	0.473747998
0.989401996	0.501685977	0.062050998
0.989154994	0.498421997	0.201853007
0.988831997	0.499606997	0.341095001
0.987112999	0.492136002	0.473179013
0.489360988	0.496199012	0.061999999
0.489095986	0.497500002	0.202106997

0.488384992	0.499608994	0.339760005
0.489715010	0.496293008	0.485547990
0.239162996	0.003116000	0.057597999
0.239181995	0.996668994	0.196200997
0.237917006	0.997542024	0.334928989
0.237114996	0.001368000	0.472995013
0.984677017	0.248674005	0.065356001
0.990701973	0.248760998	0.189881995
0.989394009	0.248565003	0.343625009
0.986424983	0.248623997	0.469090015
0.989270985	0.034141999	0.988250971
0.989521980	0.970650017	0.128059000
0.988828003	0.025722999	0.267098993
0.988557994	0.961553991	0.407090992
0.739404976	0.994180977	0.057512000
0.739161015	0.000235000	0.196174994
0.739623010	0.998748004	0.335168988
0.741119027	0.995118022	0.472487003
0.493239999	0.248843998	0.050443999
0.487244993	0.248659000	0.202516004
0.488426000	0.248382002	0.331602991
0.491499007	0.246607006	0.475217998
0.488970995	0.964868009	0.988325000
0.489650995	0.023327000	0.127983004
0.489140987	0.978955984	0.267486006
0.488411993	0.010015000	0.405575007
0.239268005	0.494352013	0.057509001
0.239224002	0.500478029	0.196230993
0.237579003	0.499776989	0.335209996
0.234418005	0.497065008	0.472680986
0.993236005	0.748860002	0.049922999
0.986990988	0.748474002	0.203820005
0.988294005	0.748432994	0.328503996
0.001420000	0.750253975	0.490709990
0.989026010	0.464002013	0.988352001
0.989449024	0.526704013	0.127982005
0.989153028	0.471347004	0.267228007
0.987707973	0.533056974	0.407099009
0.739251018	0.503273010	0.057565000
0.739064991	0.496854991	0.196251005
0.739916980	0.498887986	0.335179001
0.742452025	0.503261983	0.472389013
0.484660000	0.748763978	0.064925998
0.490839005	0.748614013	0.191603005

0.489908010	0.749132991	0.340180993
0.487127990	0.752626002	0.472314000
0.489053994	0.533357024	0.988269985
0.489282995	0.473955989	0.127990007
0.488968015	0.518319011	0.267646015
0.488703996	0.488233000	0.405270010
0.470295995	0.509850025	0.549878001
0.723783016	0.562398016	0.588213980
0.471502990	0.668556988	0.616870999
0.882677972	0.750227988	0.645026982
0.125330001	0.900044978	0.660459995
0.120765999	0.736621022	0.596063018
0.558943987	0.578516006	0.583926022
0.043531999	0.795454979	0.633422971
0.345447004	0.683165014	0.607617974
0.060630001	0.749517024	0.521619022
0.812978029	0.666738987	0.620151997

The coordinates of $LaAlO_3$-(HCO_3+HCO_3)*

La16Al16O54C2H2
1.0

7.6224999428	0.0000000000	0.0000000000
0.0000000000	7.6224999428	0.0000000000
0.0000000000	0.0000000000	28.3393993378

La	Al	O	C	H
16	16	54	2	2

Direct

0.236834005	0.251201004	0.000017000
0.252447993	0.251444012	0.137659997
0.247575000	0.251839995	0.277420998
0.257683992	0.258008003	0.417153001
0.764701009	0.250896007	0.999971986
0.748109996	0.251522005	0.137751997
0.751887977	0.251744002	0.277568996
0.741167009	0.258114010	0.417001992
0.264914989	0.751075983	0.999858022
0.249118999	0.751299977	0.137869000
0.250391006	0.752322972	0.276647002
0.257331997	0.749208987	0.417282999
0.736738980	0.751183987	0.999979973
0.751433015	0.751267016	0.138006002
0.748641014	0.752299011	0.276638001
0.738605022	0.748847008	0.416655988

0.999536991	0.998178005	0.062781997
0.999256015	0.001589000	0.202742994
0.999270976	0.001103000	0.342234999
0.999095023	0.010782000	0.472503990
0.499532014	0.003552000	0.062729001
0.499152005	0.002683000	0.202866003
0.499042988	0.001232000	0.340837985
0.499839008	0.005874000	0.474117011
0.999427974	0.503974974	0.062761001
0.999226987	0.501357973	0.202819005
0.999143004	0.503022015	0.342204005
0.998502016	0.494067013	0.475538999
0.499527991	0.498587996	0.062696002
0.499139994	0.500384986	0.202981994
0.498937994	0.503546000	0.341064990
0.499772996	0.501457989	0.483603001
0.249219999	0.009524000	0.058148000
0.249111995	0.993444979	0.197184995
0.248554006	0.005360000	0.335731000
0.248647004	0.006041000	0.473776996
0.989919007	0.251033008	0.065788001
0.005841000	0.251493007	0.190992996
0.994939983	0.252168000	0.343858004
0.998031020	0.252249986	0.470209986
0.998700023	0.035523001	0.988774002
0.999821007	0.974018991	0.128709003
0.999242008	0.027233001	0.268083006
0.999607027	0.968936026	0.407604992
0.749325991	0.991842985	0.058279999
0.749117017	0.008940000	0.197003007
0.749776006	0.998009026	0.335859001
0.751246989	0.001553000	0.473298997
0.507328987	0.250986993	0.051504001
0.490096986	0.251428992	0.202779993
0.502561986	0.252395988	0.333317995
0.501141012	0.251756996	0.475428998
0.499336004	0.968387008	0.988720000
0.499193013	0.024660001	0.128709003
0.499599993	0.984426975	0.268373996
0.499289006	0.011747000	0.406459987
0.249100000	0.492054999	0.058357999
0.249059007	0.509105027	0.196998000
0.248172000	0.498887986	0.336459011
0.245313004	0.502891004	0.473080009

0.007208000	0.751035988	0.050611001
0.990300000	0.751338005	0.204257995
0.002246000	0.751762986	0.329887986
0.008650000	0.759034991	0.488204986
0.999279022	0.466663003	0.988761008
0.998898029	0.528418005	0.128671005
0.999876976	0.475832999	0.268189996
0.997614980	0.533030987	0.408156991
0.749284029	0.509855986	0.058058999
0.749090016	0.493669987	0.197266996
0.749870002	0.506213009	0.336053014
0.753148019	0.506295979	0.473749012
0.490002006	0.751048982	0.065123998
0.505919993	0.751363993	0.192748994
0.495216995	0.752368987	0.340485007
0.498385996	0.756282985	0.473740995
0.498376995	0.534012020	0.988789976
0.499967992	0.477997005	0.128656998
0.498665988	0.519473016	0.268496007
0.500171006	0.494720012	0.406920999
0.489425987	0.505145013	0.549607992
0.627331972	0.556715012	0.620325983
0.344168007	0.589477003	0.615958989
0.919745028	0.740791023	0.630896986
0.785211980	0.504746020	0.592284977
0.050607000	0.576719999	0.572176993
0.483767986	0.548789978	0.591750979
0.926801026	0.624876022	0.601357996
0.227228999	0.584538996	0.595734000
0.049056001	0.743595004	0.521075010

The coordinates of $LaNiO_3$-O*

La16Ni16O49
1.0

7.7140002251	0.0000000000	0.0000000000
0.0000000000	7.7140002251	0.0000000000
0.0000000000	0.0000000000	28.4995002747

La Ni O
16 16 49
Direct

0.250000000	0.250000000	0.000000000
0.250000000	0.250000000	0.135340005
0.250050992	0.250070989	0.273270994

0.248741001	0.248976007	0.412492007
0.750000000	0.250000000	0.000000000
0.750000000	0.250000000	0.135340005
0.749943018	0.250059992	0.273268014
0.751205027	0.248762995	0.412481993
0.250000000	0.750000000	0.000000000
0.250000000	0.750000000	0.135340005
0.250057995	0.749933004	0.273272991
0.248900995	0.751088023	0.412508994
0.750000000	0.750000000	0.000000000
0.750000000	0.750000000	0.135340005
0.749936998	0.749944985	0.273270994
0.751029015	0.751307011	0.412492007
0.000000000	0.000000000	0.067670003
0.000000000	0.000000000	0.202999994
0.000005000	0.999997973	0.338988006
0.999997973	0.000026000	0.476577014
0.500000000	0.000000000	0.067670003
0.500000000	0.000000000	0.202999994
0.499978006	0.000051000	0.339020014
0.499940008	0.999864995	0.476673990
0.000000000	0.500000000	0.067670003
0.000000000	0.500000000	0.202999994
0.999943972	0.500018001	0.339017987
0.000167000	0.500065982	0.476678997
0.500000000	0.500000000	0.067670003
0.500000000	0.500000000	0.202999994
0.500033021	0.499980986	0.340079993
0.499897987	0.500081003	0.478051007
0.000000000	0.000000000	0.000000000
0.000000000	0.000000000	0.135340005
0.999966979	0.000012000	0.270696998
0.999940991	0.000056000	0.405467987
0.250000000	0.000000000	0.067670003
0.250000000	0.000000000	0.202999994
0.250156999	0.000012000	0.335970998
0.249891996	0.999769986	0.470306993
0.000000000	0.250000000	0.067670003
0.000000000	0.250000000	0.202999994
0.999993980	0.250169009	0.336003006
0.000370000	0.249939993	0.470207989
0.500000000	0.000000000	0.000000000
0.500000000	0.000000000	0.135340005
0.500018001	0.999921978	0.270720989

0.499969989	0.000413000	0.405351013
0.750000000	0.000000000	0.067670003
0.750000000	0.000000000	0.202999994
0.749831975	0.000038000	0.336021990
0.750074029	0.999725997	0.470212013
0.500000000	0.250000000	0.067670003
0.500000000	0.250000000	0.202999994
0.500034988	0.249714002	0.336668015
0.499888003	0.245971993	0.467795998
0.000000000	0.500000000	0.000000000
0.000000000	0.500000000	0.135340005
0.000098000	0.500006020	0.270722985
0.999490976	0.500007987	0.405351996
0.250000000	0.500000000	0.067670003
0.250000000	0.500000000	0.202999994
0.249714002	0.499962986	0.336760014
0.246378005	0.500151992	0.467873007
0.000000000	0.750000000	0.067670003
0.000000000	0.750000000	0.202999994
0.999983013	0.749845982	0.335972995
0.000294000	0.750136018	0.470286995
0.500000000	0.500000000	0.000000000
0.500000000	0.500000000	0.135340005
0.499859005	0.500111997	0.270747989
0.500099003	0.499933004	0.408542007
0.750000000	0.500000000	0.067670003
0.750000000	0.500000000	0.202999994
0.750262022	0.499985993	0.336654007
0.754100025	0.500119984	0.467808008
0.500000000	0.750000000	0.067670003
0.500000000	0.750000000	0.202999994
0.500036001	0.750277996	0.336741000
0.499855995	0.753651023	0.467848003
0.509037018	0.492931008	0.539906979

The coordinates of $LaNiO_3$-OH*

La16Ni16O49H1
1.0

7.7140002251	0.0000000000	0.0000000000
0.0000000000	7.7140002251	0.0000000000
0.0000000000	0.0000000000	28.4995002747

La	Ni	O	H
16	16	49	1

Direct

0.250000000	0.250000000	0.000000000
0.250000000	0.250000000	0.135340005
0.250059009	0.250117004	0.273111999
0.249032006	0.250477999	0.412299007
0.750000000	0.250000000	0.000000000
0.750000000	0.250000000	0.135340005
0.750155985	0.249946997	0.273149014
0.751295984	0.249785006	0.412061006
0.250000000	0.750000000	0.000000000
0.250000000	0.750000000	0.135340005
0.250079006	0.749984980	0.273128003
0.249044001	0.749491990	0.412344009
0.750000000	0.750000000	0.000000000
0.750000000	0.750000000	0.135340005
0.750092983	0.750141978	0.273142993
0.751257002	0.750316978	0.411985993
0.000000000	0.000000000	0.067670003
0.000000000	0.000000000	0.202999994
0.000225000	0.000025000	0.338804007
0.999576986	0.999945998	0.476110995
0.500000000	0.000000000	0.067670003
0.500000000	0.000000000	0.202999994
0.499989003	0.000134000	0.338952005
0.500419974	0.999867022	0.476635009
0.000000000	0.500000000	0.067670003
0.000000000	0.500000000	0.202999994
0.000452042	0.500016987	0.338910997
0.998737991	0.500050008	0.476332992
0.500000000	0.500000000	0.067670003
0.500000000	0.500000000	0.202999994
0.500315011	0.500078976	0.339578003
0.501236975	0.500084996	0.478026003
0.000000000	0.000000000	0.000000000
0.000000000	0.000000000	0.135340005
0.999981999	0.000004000	0.270615995
0.000862002	0.000018000	0.405081987
0.250000000	0.000000000	0.067670003
0.250000000	0.000000000	0.202999994
0.250003010	0.999983013	0.335873008
0.249493003	0.000046968	0.470169008
0.000000000	0.250000000	0.067670003
0.000000000	0.250000000	0.202999994
0.999830008	0.249971002	0.336080998

0.000770000	0.249593005	0.470093012
0.500000000	0.000000000	0.000000000
0.500000000	0.000000000	0.135340005
0.500023007	0.999980986	0.270613015
0.499547988	0.000533000	0.405600011
0.750000000	0.000000000	0.067670003
0.750000000	0.000000000	0.202999994
0.750178993	0.000089000	0.335978001
0.750505984	0.000062943	0.469303012
0.500000000	0.250000000	0.067670003
0.500000000	0.250000000	0.202999994
0.500280976	0.249834999	0.336497992
0.499323994	0.246865004	0.468026012
0.000000000	0.500000000	0.000000000
0.000000000	0.500000000	0.135340005
0.000508000	0.499976009	0.270716012
0.003834963	0.499924004	0.405328006
0.250000000	0.500000000	0.067670003
0.250000000	0.500000000	0.202999994
0.250097990	0.500027001	0.336358994
0.249273002	0.499624014	0.469830006
0.000000000	0.750000000	0.067670003
0.000000000	0.750000000	0.202999994
0.999889016	0.750059009	0.336091995
0.000849000	0.750382006	0.470021993
0.500000000	0.500000000	0.000000000
0.500000000	0.500000000	0.135340005
0.499395996	0.500311971	0.271227002
0.498275012	0.499763012	0.406899989
0.750000000	0.500000000	0.067670003
0.750000000	0.500000000	0.202999994
0.750562012	0.499992996	0.336457998
0.751875997	0.500108004	0.467112988
0.500000000	0.750000000	0.067670003
0.500000000	0.750000000	0.202999994
0.500549018	0.750389993	0.336686999
0.499484986	0.753148019	0.467821985
0.497808993	0.498686999	0.543075979
0.373842001	0.489102989	0.549960971

The coordinates of $LaNiO_3$-OOH*

La16Ni16O50H1
1.0

7.7140002251	0.0000000000	0.0000000000
0.0000000000	7.7140002251	0.0000000000
0.0000000000	0.0000000000	28.4995002747

La	Ni	O	H
16	16	50	1

Direct

0.250000000	0.250000000	0.000000000
0.250000000	0.250000000	0.135340005
0.250021994	0.249966994	0.273057014
0.250766993	0.250734001	0.412528008
0.750000000	0.250000000	0.000000000
0.750000000	0.250000000	0.135340005
0.750162005	0.250010997	0.273097008
0.749137998	0.250481009	0.412194014
0.250000000	0.750000000	0.000000000
0.250000000	0.750000000	0.135340005
0.250090986	0.750050008	0.273066998
0.250692010	0.749323010	0.412461996
0.750000000	0.750000000	0.000000000
0.750000000	0.750000000	0.135340005
0.750088990	0.750005007	0.273113012
0.749243975	0.749607027	0.412153006
0.000000000	0.000000000	0.067670003
0.000000000	0.000000000	0.202999994
0.000229000	0.000020000	0.338856995
0.999855995	0.000041962	0.475719988
0.500000000	0.000000000	0.067670003
0.500000000	0.000000000	0.202999994
0.499967009	0.000120000	0.338755995
0.500240982	0.000303984	0.475672007
0.000000000	0.500000000	0.067670003
0.000000000	0.500000000	0.202999994
0.000245000	0.499989986	0.338766992
0.999553025	0.500047982	0.475621015
0.500000000	0.500000000	0.067670003
0.500000000	0.500000000	0.202999994
0.499893993	0.500020027	0.339262009
0.500196993	0.500252008	0.479719996
0.000000000	0.000000000	0.000000000
0.000000000	0.000000000	0.135340005
0.999948978	0.000017000	0.270603001
0.001330000	0.999940991	0.405144006
0.250000000	0.000000000	0.067670003
0.250000000	0.000000000	0.202999994

0.250104994	0.999961019	0.335554987
0.250362009	0.000088000	0.470443010
0.000000000	0.250000000	0.067670003
0.000000000	0.250000000	0.202999994
0.999958992	0.250014007	0.335714996
0.000010000	0.250297010	0.470086008
0.500000000	0.000000000	0.000000000
0.500000000	0.000000000	0.135340005
0.500240982	0.999695003	0.270577997
0.499417990	0.001220000	0.404976994
0.750000000	0.000000000	0.067670003
0.750000000	0.000000000	0.202999994
0.750086010	0.000029000	0.335902005
0.749800026	0.000066000	0.469698012
0.500000000	0.250000000	0.067670003
0.500000000	0.250000000	0.202999994
0.500360012	0.250230998	0.336041987
0.500068009	0.248220995	0.468818992
0.000000000	0.500000000	0.000000000
0.000000000	0.500000000	0.135340005
0.999453008	0.499998003	0.270583987
0.002661000	0.500052989	0.404987991
0.250000000	0.500000000	0.067670003
0.250000000	0.500000000	0.202999994
0.250198990	0.500090003	0.335758001
0.247235999	0.500145972	0.469033003
0.000000000	0.750000000	0.067670003
0.000000000	0.750000000	0.202999994
0.999804974	0.749989986	0.335738003
0.999837995	0.749734998	0.470072001
0.500000000	0.500000000	0.000000000
0.500000000	0.500000000	0.135340005
0.500751972	0.500497997	0.270644993
0.497750998	0.499660999	0.405851990
0.750000000	0.500000000	0.067670003
0.750000000	0.500000000	0.202999994
0.749945998	0.499949992	0.336645991
0.752233028	0.499908000	0.468153000
0.500000000	0.750000000	0.067670003
0.500000000	0.750000000	0.202999994
0.500503004	0.749918997	0.336380005
0.499969006	0.752519011	0.468533009
0.537086010	0.374210000	0.573657990
0.490258992	0.524339974	0.549997985

0.526414990	0.411361992	0.606531978

The coordinates of $LaCuO_3$-O*

La16Cu16O49
1.0

7.7432999611	0.0000000000	0.0000000000
0.0000000000	7.7432999611	0.0000000000
0.0000000000	0.0000000000	28.5508003235

La	Cu	O
16	16	49

Direct

0.250000000	0.250000000	0.000000000
0.250000000	0.250000000	0.135609999
0.249714002	0.251100987	0.273777992
0.248440996	0.248846993	0.413592994
0.750000000	0.250000000	0.000000000
0.750000000	0.250000000	0.135609999
0.750110984	0.249724001	0.273687989
0.751003981	0.248830006	0.414079010
0.250000000	0.750000000	0.000000000
0.250000000	0.750000000	0.135609999
0.250674993	0.749234974	0.273833007
0.247592002	0.752381980	0.413082987
0.750000000	0.750000000	0.000000000
0.750000000	0.750000000	0.135609999
0.748944998	0.750365019	0.273770005
0.751065016	0.751412988	0.413650006
0.000000000	0.000000000	0.067800000
0.000000000	0.000000000	0.203410000
0.999705970	0.000190000	0.338479996
0.999854028	0.000030000	0.478419989
0.500000000	0.000000000	0.067800000
0.500000000	0.000000000	0.203410000
0.499830991	0.000745000	0.338611990
0.499282002	0.999190986	0.478300989
0.000000000	0.500000000	0.067800000
0.000000000	0.500000000	0.203410000
0.999288976	0.500104010	0.338589996
0.000814000	0.500546992	0.478285015
0.500000000	0.500000000	0.067800000
0.500000000	0.500000000	0.203410000
0.500231028	0.499956995	0.340137988
0.497276008	0.502462029	0.479267001

0.000000000	0.000000000	0.000000000
0.000000000	0.000000000	0.135609999
0.998169005	0.001178000	0.271019012
0.000537000	0.999638021	0.403560996
0.250000000	0.000000000	0.067800000
0.250000000	0.000000000	0.203410000
0.249761999	0.000323000	0.335388988
0.248125002	0.999861002	0.469045013
0.000000000	0.250000000	0.067800000
0.000000000	0.250000000	0.203410000
0.999410987	0.250203997	0.335705012
0.000407000	0.248823002	0.469134003
0.500000000	0.000000000	0.000000000
0.500000000	0.000000000	0.135609999
0.501277983	0.996752977	0.271147996
0.498672992	0.005152000	0.403490007
0.750000000	0.000000000	0.067800000
0.750000000	0.000000000	0.203410000
0.749772012	0.999611020	0.335922986
0.751039028	0.999534011	0.469112992
0.500000000	0.250000000	0.067800000
0.500000000	0.250000000	0.203410000
0.499929994	0.250842988	0.334876001
0.499399990	0.239766002	0.469208002
0.000000000	0.500000000	0.000000000
0.000000000	0.500000000	0.135609999
0.004081000	0.499159008	0.271124005
0.994072020	0.500855982	0.403488010
0.250000000	0.500000000	0.067800000
0.250000000	0.500000000	0.203410000
0.250380993	0.499859005	0.337408990
0.240989000	0.501205027	0.467310995
0.000000000	0.750000000	0.067800000
0.000000000	0.750000000	0.203410000
0.000054000	0.750114024	0.335465997
0.000032000	0.751852989	0.469056010
0.500000000	0.500000000	0.000000000
0.500000000	0.500000000	0.135609999
0.496192008	0.503543019	0.271474987
0.505540013	0.495000005	0.408387989
0.750000000	0.500000000	0.067800000
0.750000000	0.500000000	0.203410000
0.749264002	0.500276983	0.334533006
0.760221004	0.500428975	0.469298989

0.500000000	0.750000000	0.067800000
0.500000000	0.750000000	0.203410000
0.499561012	0.749760985	0.337262988
0.498755008	0.758969009	0.467408001
0.519405007	0.481456012	0.541299999

The coordinates of $LaCuO_3$-OH*

La16Cu16O49H1

1.0

7.7432999611	0.0000000000	0.0000000000
0.0000000000	7.7432999611	0.0000000000
0.0000000000	0.0000000000	28.5508003235

La	Cu	O	H
16	16	49	1

Direct

0.250000000	0.250000000	0.000000000
0.250000000	0.250000000	0.135609999
0.250631988	0.249574006	0.273844004
0.249363005	0.249063000	0.413953006
0.750000000	0.250000000	0.000000000
0.750000000	0.250000000	0.135609999
0.750660002	0.250550002	0.273889005
0.751959026	0.248495996	0.413365990
0.250000000	0.750000000	0.000000000
0.250000000	0.750000000	0.135609999
0.250916004	0.750362992	0.273869991
0.249662995	0.749988019	0.414005995
0.750000000	0.750000000	0.000000000
0.750000000	0.750000000	0.135609999
0.750443995	0.749423027	0.273900002
0.752005994	0.750952005	0.413298994
0.000000000	0.000000000	0.067800000
0.000000000	0.000000000	0.203410000
0.000710000	0.999935985	0.338665992
0.000098000	0.999575019	0.478588998
0.500000000	0.000000000	0.067800000
0.500000000	0.000000000	0.203410000
0.500505984	0.999947011	0.338721007
0.501103997	0.999444008	0.478902012
0.000000000	0.500000000	0.067800000
0.000000000	0.500000000	0.203410000
0.001401000	0.500001013	0.338739991
0.999085009	0.499689996	0.478845000

0.500000000	0.500000000	0.067800000
0.500000000	0.500000000	0.203410000
0.500841022	0.499859005	0.340126991
0.500997007	0.499451995	0.478738010
0.000000000	0.000000000	0.000000000
0.000000000	0.000000000	0.135609999
0.001734000	0.000419000	0.271111995
0.000055000	0.999722004	0.403744996
0.250000000	0.000000000	0.067800000
0.250000000	0.000000000	0.203410000
0.250732988	0.000016000	0.335893005
0.249310002	0.999561012	0.469404995
0.000000000	0.250000000	0.067800000
0.000000000	0.250000000	0.203410000
0.000521000	0.250064999	0.335839003
0.000231000	0.248809993	0.469430000
0.500000000	0.000000000	0.000000000
0.500000000	0.000000000	0.135609999
0.498851001	0.998876989	0.271169990
0.500994027	0.000780000	0.403595001
0.750000000	0.000000000	0.067800000
0.750000000	0.000000000	0.203410000
0.750524998	0.999909997	0.335391015
0.751740992	0.999747992	0.469107002
0.500000000	0.250000000	0.067800000
0.500000000	0.250000000	0.203410000
0.501042008	0.250712991	0.336075991
0.500787020	0.239556998	0.467844993
0.000000000	0.500000000	0.000000000
0.000000000	0.500000000	0.135609999
0.997584999	0.499545991	0.271209002
0.006193000	0.500138998	0.403618008
0.250000000	0.500000000	0.067800000
0.250000000	0.500000000	0.203410000
0.251830012	0.499839991	0.335332006
0.242835999	0.499262005	0.470155001
0.000000000	0.750000000	0.067800000
0.000000000	0.750000000	0.203410000
0.000634000	0.749840975	0.335501999
0.000284000	0.750572026	0.469368011
0.500000000	0.500000000	0.000000000
0.500000000	0.500000000	0.135609999
0.502582014	0.500526011	0.271468014
0.496601999	0.498950005	0.409164995

0.750000000	0.500000000	0.067800000
0.750000000	0.500000000	0.203410000
0.750145018	0.500051975	0.337267995
0.758754015	0.499769002	0.466903001
0.500000000	0.750000000	0.067800000
0.500000000	0.750000000	0.203410000
0.501088977	0.749077976	0.336717010
0.501159012	0.759516001	0.467586011
0.496470004	0.498136014	0.542997003
0.372844011	0.486690998	0.549583018

The coordinates of $LaCuO_3$-OOH*

La16Cu16O50H1
1.0

7.7432999611	0.0000000000	0.0000000000
0.0000000000	7.7432999611	0.0000000000
0.0000000000	0.0000000000	28.5508003235

La	Cu	O	H
16	16	50	1

Direct

0.250000000	0.250000000	0.000000000
0.250000000	0.250000000	0.135609999
0.250432014	0.249599993	0.273815006
0.249879003	0.248978004	0.414038986
0.750000000	0.250000000	0.000000000
0.750000000	0.250000000	0.135609999
0.750285983	0.250694990	0.273865014
0.750980973	0.248768002	0.413791001
0.250000000	0.750000000	0.000000000
0.250000000	0.750000000	0.135609999
0.250735015	0.750182986	0.273824006
0.249890998	0.750801027	0.413962990
0.750000000	0.750000000	0.000000000
0.750000000	0.750000000	0.135609999
0.749895990	0.749141991	0.273891985
0.751016974	0.751007974	0.413729995
0.000000000	0.000000000	0.067800000
0.000000000	0.000000000	0.203410000
0.000332000	0.999942005	0.338770986
0.000332000	0.999875009	0.478527993
0.500000000	0.000000000	0.067800000
0.500000000	0.000000000	0.203410000
0.500362992	0.999970973	0.338815987

0.500706971	0.000030000	0.478231996
0.000000000	0.500000000	0.067800000
0.000000000	0.500000000	0.203410000
0.000627000	0.499945998	0.338804007
0.000113000	0.499967992	0.478067994
0.500000000	0.500000000	0.067800000
0.500000000	0.500000000	0.203410000
0.500145972	0.499819994	0.339969993
0.500697017	0.499743998	0.478184015
0.000000000	0.000000000	0.000000000
0.000000000	0.000000000	0.135609999
0.001711000	0.000511000	0.271270007
0.999704003	0.999809980	0.403793007
0.250000000	0.000000000	0.067800000
0.250000000	0.000000000	0.203410000
0.250573009	0.999900997	0.336030006
0.249960005	0.999760985	0.469581991
0.000000000	0.250000000	0.067800000
0.000000000	0.250000000	0.203410000
0.000465000	0.250209987	0.335635990
0.000235000	0.249185994	0.469496995
0.500000000	0.000000000	0.000000000
0.500000000	0.000000000	0.135609999
0.498760998	0.998839974	0.271266997
0.501465976	0.000812000	0.403724015
0.750000000	0.000000000	0.067800000
0.750000000	0.000000000	0.203410000
0.750119984	0.999960005	0.335435987
0.751031995	0.999736011	0.469630986
0.500000000	0.250000000	0.067800000
0.500000000	0.250000000	0.203410000
0.500339985	0.250950992	0.335850000
0.500485003	0.245242000	0.468620002
0.000000000	0.500000000	0.000000000
0.000000000	0.500000000	0.135609999
0.997219026	0.499673009	0.271281004
0.003762000	0.500111997	0.403726995
0.250000000	0.500000000	0.067800000
0.250000000	0.500000000	0.203410000
0.251298010	0.499924004	0.335231006
0.244618997	0.500060976	0.469036996
0.000000000	0.750000000	0.067800000
0.000000000	0.750000000	0.203410000
0.000144000	0.749740005	0.335509986

0.000130000	0.750588000	0.469518006
0.500000000	0.500000000	0.000000000
0.500000000	0.500000000	0.135609999
0.502653003	0.500703990	0.271019995
0.497848004	0.499199986	0.407296002
0.750000000	0.500000000	0.067800000
0.750000000	0.500000000	0.203410000
0.749427974	0.499821007	0.337190986
0.755703986	0.499967992	0.468149006
0.500000000	0.750000000	0.067800000
0.500000000	0.750000000	0.203410000
0.500367999	0.748794973	0.336409986
0.500513017	0.754904985	0.468371004
0.536710978	0.373358011	0.573275983
0.489333987	0.522431016	0.550044000
0.522939026	0.407530993	0.606266975

References

[1] Kuttassery, F.; Mathew, S.; Sagawa, S.; Remello, S. N.; Thomas, A.; Yamamoto, D.; Onuki, S.; Nabetani, Y.; Tachibana, H.; Inoue, H. One Electron-Initiated Two-Electron Oxidation of Water by Aluminum Porphyrins with Earth's Most Abundant Metal. *ChemSusChem* **2017**, *10* (9), 1909– 1915.

[2] Ohsaki, Y.; Thomas, A.; Kuttassery, F.; Mathew, S.; Remello, S. N.; Shimada, T.; Ishida, T.; Takagi, S.; Tachibana, H.; Inoue, H. Two-Electron Oxidation of Water to Form Hydrogen Peroxide Initiated by One-Electron Oxidation of Tin (IV)-Porphyrins. *J. Photochem. Photobiol., A* **2020**, *401*, 112732.
